# Tunable Hybrid Hydrogels of Alginate and Cell‐Derived dECM to Study the Impact of Matrix Alterations on Epithelial‐to‐Mesenchymal Transition

**DOI:** 10.1002/adhm.202401032

**Published:** 2024-09-09

**Authors:** P. Barros da Silva, Xiaoyu Zhao, Sílvia J. Bidarra, Diana S. Nascimento, Vernon LaLone, Bianca N. Lourenço, Joana Paredes, Molly M. Stevens, C. C. Barrias

**Affiliations:** ^1^ i3S – Instituto de Investigação e Inovação em Saúde Universidade do Porto Rua Alfredo Allen 208 Porto 4200‐135 Portugal; ^2^ INEB – Instituto de Engenharia Biomédica Universidade do Porto Porto 4200‐135 Portugal; ^3^ FEUP – Faculdade de Engenharia da Universidade do Porto Porto 4200‐135 Portugal; ^4^ Department of Bioengineering Imperial College London Exhibition Rd London SW7 2AZ UK; ^5^ Institute of Biomedical Engineering Imperial College London Exhibition Rd London SW7 2AZ UK; ^6^ ICBAS – Instituto de Ciências Biomédicas Abel Salazar Universidade do Porto Porto 4200‐135 Portugal; ^7^ Department of Materials Imperial College London Exhibition Rd London SW7 2AZ UK; ^8^ FMUP – Faculdade de Medicina da Universidade do Porto Porto 4200‐319 Portugal; ^9^ IPATIMUP – Instituto de Patologia e Imunologia Molecular da Universidade do Porto Porto 4200‐135 Portugal

**Keywords:** cell‐derived decellularized ECM, defined hydrogel, tumor microenvironment, tunable hydrogel

## Abstract

Epithelial‐to‐mesenchymal transition (EMT) is crucial for tumor progression, being linked to alterations in the extracellular matrix (ECM). Understanding the ECM's role in EMT can uncover new therapeutic targets, yet replicating these interactions in vitro remains challenging. It is shown that hybrid hydrogels of alginate (ALG) and cell‐derived decellularized ECM (dECM), with independently tunable composition and stiffness, are useful 3D‐models to explore the impact of the breast tumor matrix on EMT. Soft RGD‐ALG hydrogels (200 Pa), used as neutral bulk material, supported mammary epithelial cells morphogenesis without spontaneous EMT, allowing to define the gene, protein, and biochemical profiles of cells at different TGFβ1‐induced EMT states. To mimic the breast tumor composition, dECM from TGFβ1‐activated fibroblasts (adECM) are generated, which shows upregulation of tumor‐associated proteins compared to ndECM from normal fibroblasts. Using hybrid adECM‐ALG hydrogels, it is shown that the presence of adECM induces partial EMT in normal epithelial cells, and amplifes TGF‐β1 effects compared to ALG and ndECM‐ALG. Increasing the hydrogel stiffness to tumor‐like levels (2.5 kPa) have a synergistic effect, promoting a more evident EMT. These findings shed light on the complex interplay between matrix composition and stiffness in EMT, underscoring the utility of dECM‐ALG hydrogels as a valuable in vitro platform for cancer research.

## Graphical abstract

This study introduces a novel 3D model to explore how ECM properties impact EMT in breast cancer. By combining mechanically‐tunable alginate hydrogels with tumoral‐like decellularized ECM (adECM), the platform successfully recreates key aspects of the TME. It is demonstrated that adECM induces partial EMT in epithelial cells, enhanced by TGF‐β1 and modulated by matrix stiffness, offering new insights into tumor progression mechanisms.

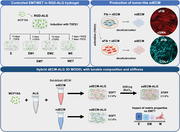

## 1 Introduction

Epithelial‐to‐mesenchymal (EMT) and mesenchymal‐to‐epithelial transitions (MET) are dynamic biological processes involving phenotypic switches between epithelial and mesenchymal‐like states.^[^ ^1^ ^]^ In cancer, EMT results in the downregulation of epithelial features, such as loss of polarity and intercellular adhesions, and the acquisition of mesenchymal ones, including increased migratory and invasive potential, which are crucial for metastatic dissemination. In addition, MET facilitates the establishment of secondary tumors at metastatic sites.^[^ ^2^ ^]^ EMT is triggered by signals from the tumor microenvironment (TME), which includes various cell types, soluble mediators, and the extracellular matrix (ECM).^[^ ^3^, ^4^ ^]^ During cancer progression, cancer‐associated fibroblasts (CAFs) contribute to the production of an increased amount of ECM with altered composition and stiffness.^[^ ^5^ ^]^ This aberrant ECM creates a microenvironment conducive to tumor growth and dissemination, facilitating adaptive changes in neoplastic cells, particularly through EMT.^[^ ^6^, ^7^ ^]^ Therefore, understanding the interaction between matrix properties and tumor cells may uncover new mechanisms underlying cancer progression and lead to EMT‐targeted therapeutic approaches.^[^ ^8^ ^]^

While several in vitro models of EMT have been described,^[^ ^9^, ^10^, ^11^, ^12^ ^]^ 3D systems specifically designed to explore the impact of matrix properties on this complex biological program remain scarce. Recent advancements in materials engineering have enabled the creation of artificial ECM‐like hydrogels with well‐defined and reproducible properties. These hydrogels provide biologically neutral backgrounds for custom modifications with bioactive moieties and can be crosslinked using a variety of techniques. This flexibility allows for the creation of 3D matrices with individually tunable biochemical and biophysical properties, offering valuable platforms for systematic investigation of matrix effects on cell behavior.^[^ ^9^, ^10^ ^]^ The successful induction of the EMT program on epithelial cells cultured in chemically defined hydrogels, namely of alginate or polyethylene‐glycol (PEG), has been previously reported by us and other groups.^[^ ^9^, ^10^ ^]^ Still, these materials fail to emulate the rich composition of the tumor ECM. Alternatively, ECM‐derived hydrogels, such Matrigel from mouse osteosarcomas, have also been used.^[^ ^13^ ^]^ These are rich in basement membrane components and growth factors, providing a more biologically relevant environment for epithelial cell culture. However, despite being the gold standard in the field, Matrigel has an undefined composition and high batch‐to‐batch variability, and its mechanical properties are not customizable. These limitations restrict its applicability as an in vitro model for mechanistic studies on cell‐matrix interactions.

Hybrid systems that combine artificial and ECM‐derived hydrogels, such as alginate with Matrigel or collagen, offer a promising alternative by providing both tunability and enhanced physiological relevance.^[^ ^14^, ^15^, ^16^ ^]^ However, these materials still lack the specific compositional features unique to different tissues and pathological conditions. The use of decellularized (dECM) obtained from patient‐derived tumors provides a relevant option, but its use is hampered by the limited availability of human samples and the high variability between individuals and tumor types.^[^ ^17^ ^]^ In this context, cell‐derived dECM represents a valuable yet relatively unexplored alternative.^[^ ^18^, ^19^ ^]^ By selecting the appropriate cell type, it is possible to produce a dECM in vitro and under well‐controlled conditions, which captures the composition of a specific tissue or organ. Furthermore, cells can be selectively primed to generate an altered dECM, such as the tumoral ECM, that can be directly compared with its normal counterpart, providing a significant advantage.^[^ ^18^, ^19^ ^]^

In this study, we introduce hybrid hydrogels composed of alginate (ALG) and cell‐derived dECM as a novel 3D model to investigate the effects of the breast tumor matrix on EMT in normal mammary epithelial MCF10A cells. This model uses ALG as a neutral bulk material, which supports epithelial morphogenesis without inducing EMT, while also allowing for on‐demand modulation of the hydrogel's stiffness through external ionic crosslinking. The cell‐derived ECM was obtained from mammary fibroblast cultures, then decellularized, solubilized, and mixed with ALG. To produce an abnormal ECM that would mimic the breast tumor matrix composition (adECM), we used TGF‐β1‐activated fibroblasts (adECM), which adopt a CAF‐like phenotype in vitro.^[^ ^20^ ^]^ Untreated fibroblasts were used to obtain the normal ECM counterpart (ndECM). We thoroughly characterized both individual components and the hybrid hydrogels, and then used the dECM‐ALG 3D model to show how variations in matrix composition (i.e., adECM vs ndECM) and stiffness (i.e., stiff vs soft) may induce different degrees of EMT in MCF10A cells.

## 2 Experimental Section

### 2.1 Cell Sources and Maintenance

Human mammary fibroblasts (ScienceCell Research Laboratories) were maintained in high glucose Fibroblast Medium (Innoprot), consisting of basal medium supplemented with 2% fetal bovine serum, 1% v/v fibroblast growth supplement and 1% v/v Pen/Strep. Fibroblasts were used between passages 4 and 10. Human mammary epithelial cells (MCF10A cell line, ATCC) were maintained in Mammary Epithelial Cell Growth Medium (Labclinics, SA), composed of basal medium supplemented with 0.4% v/v bovine pituitary extract, 10 ng mL^−1^ EGF, 0.5 µg mL^−1^ hydrocortisone, 100 ng mL^−1^ cholera toxin, 10 ng mL^−1^ human insulin, and 1% v/v Pen/Strep. MCF10A cells were used between passages 5 and 10.

### 2.2 Chemical Conjugation of Alginate with RGD Peptides

Ultrapure sodium alginate (PRONOVA UP LVG, Novamatrix, FMC Biopolymers) with high guluronic acid content (≈70%) and molecular weight ≈200 kDa was covalent grafted with RGD [(glycine)4‐arginine‐glycine‐aspartic acid‐serine‐proline, GenScript) peptides by aqueous carbodiimide chemistry as described previously.^[^ ^21^, ^22^ ^]^ Briefly, an ALG solution at 1 wt.% in MES buffer (0.1 M 2‐(N‐morpholino)‐ethanesulfonic acid, 0.3 M NaCl, pH 6.5) was prepared and stirred overnight (ON) at room temperature (RT). Then, N‐hydroxy‐sulfosuccinimide (Sulfo‐NHS, Pierce) and 1‐ethyl‐(dimethylaminopropyl)‐carbodiimide (EDC, Sigma, 27.4 mg per gram of ALG) were sequentially added at a molar ratio of 1:2, followed by 70 mg of RGD peptide (Genscript) per gram of ALG. After stirring for ≈20 h, the reaction was quenched with hydroxylamine (Sigma) and the solution was dialyzed against deionized water for 3 days (MWCO 3500). After purification with charcoal, RGD‐ALG was lyophilized and stored at −20 °C until further use. The final derivative contained 30 mg peptide per gram of ALG as quantified by the BCA Protein Assay (Pierce).

### 2.3 3D Culture of MCF10A in RGD‐Alginate Hydrogel and EMT/MET Induction

**Figure 1:**
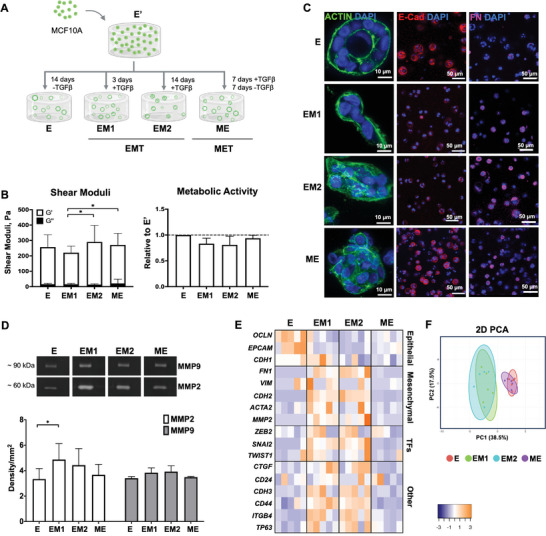
RGD‐ALG hydrogel facilitates epithelial morphogenesis and enables EMT/MET induction. A) Schematic representation of the experimental setting: human mammary epithelial cells (MCF10A) were cultured in RGD‐ALG hydrogels and four different EMT/MET states were generated by maintaining cells in the presence/absence of TGF‐β1: E (epithelial‐like state), EM1 and EM2 (mesenchymal‐like E‐to‐M states) and ME (a reversed M‐to‐E state). Created with BioRender.com. B‐i) Viscoelastic properties (G’ – elastic and G” – viscous components of the shear moduli) of 1 wt.% cell‐laden RGD‐ALG hydrogels. B‐ii) Metabolic activity (resazurin assay) of cells in the different states relative to control cultures (i.e., untreated E cells maintained in culture for the same time). Data is presented as mean ± standard deviation, n  =  4 individual experiments, *p < 0.05. C) Representative immunofluorescence images of whole‐mounted 3D cultures at E, EM1, EM2 and ME states, stained for F‐actin (green), E‐cadherin (red, E‐marker) and fibronectin (magenta, M‐marker). D) Gelatine zymography analysis of MMPs activity in 3D culture supernatants. Data is presented as mean ± standard deviation, n  =  3 individual experiments, *p < 0.05. E) Heatmap of mRNA expression profiles of the different EMT/MET states. Data displayed as absolute values normalized to GAPDH (n  =  5 individual experiments: statistical differences and p‐values are depicted in Figure S2, Supporting Information). F) Principal component analysis (PCA) using gene datasets of E, EM1, EM2 and ME populations.

Human mammary MCF10A cells were embedded in 1 wt.% RGD‐ALG hydrogels (200 µM RGD, ≈200 Pa), a formulation previously optimized by us.^[^ ^10^, ^21^, ^22^ ^]^ To establish 3D cultures, cells (5 × 10^6^ cells mL^−1^) were combined with sterile‐filtered (0.22 µm) RGD‐ALG in 0.9 wt.% NaCl. Sterile CaCO_3_ (Fluka, molar CaCO_3_/COOH = 1.7) and a fresh glucone delta‐lactone (GDL, Sigma‐Aldrich, molar CaCO_3_/GDL = 0.125) solution were then added to trigger gelation.^[^ ^10^, ^21^, ^22^ ^]^ The gel‐precursor solutions were pipetted (20 µL) onto Teflon plates, and hydrogel discs were casted between two plates separated by 750 µm‐height spacers. After gelation (15 min) cell‐laden hydrogels were transferred to non‐treated 48‐well culture plates with culture medium. 3D cultures were maintained in Mammary Epithelial Cell Growth Medium, which was changed every other day. For EMT induction (Figure 1A) epithelial MCF10A cells (E’) were maintained in culture for 3 days (EM1) or 14 days (EM2) in medium supplemented with 16 ng mL^−1^ of TGF‐β1 to generate mesenchymal‐like states. To induce MET, E’ cells were maintained in media supplemented with TGF‐β1 for 7 days (to induce a mesenchymal transition) and then in medium with no TGF‐β1 for 7 days for partial reversion (ME). Control epithelial cells (E) were maintained in culture for 14 days in medium with no TGF‐β1.

### 2.4 Metabolic Activity

The metabolic activity of 3D cultured cells was assessed using the resazurin assay. Cell‐laden hydrogel discs were incubated with 20% v/v resazurin (Sigma‐Aldrich) solution in DMEM/F12 GlutaMax (Gibco) for 2 h at 37 °C. Thereafter, 200 µL of each sample were transferred into a 96‐well black plate with clear bottom and fluorescence was measured (Ex = 530 nm, Em = 590 nm) in a Synergy MxTM (BioTek) reader.

### 2.5 Oscillation Rheometry

Rheological measurements were carried out using a Kinexus Pro rheometer (Malvern). Cell‐laden hydrogel discs were assayed using a plate‐and‐plate geometry (4 mm diameter, sandblasted surfaces) and were compressed to 20% of their original thickness to avoid slippage. A solvent trap was used to avoid sample drying. All measurements were performed at 37 °C (Peltier system). Stress sweeps (0.1 Hz) were first performed to determine the LVR. Frequency sweeps (0.01–1 Hz) were then performed within the LVR. The values of the shear moduli (G′ and G″) and phase angle, were obtained at a frequency of 0.1 Hz. Samples were analyzed in triplicate for each experiment.

### 2.6 Zymography Analysis

The activity of secreted matrix metalloproteinases (MMPs) from 3D cultures of cells at different EMT/MET states was assessed using gelatin zymography. 3D culture supernatants were collected after incubating cell‐laden hydrogel discs (3 biological replicates) in DMEM/F‐12 with GlutaMax (Gibco) and 1% v/v Pen/Strep (Gibco) for 24 h. Conditioned media were centrifuged to remove cell debris and then loaded into gelatin‐SDS polyacrylamide gels. Sample volumes were adjusted to ensure equivalent total protein content per sample. The gel was run in 1× Tris‐Glycine SDS running buffer at 60 V using the Mini Protean Tetra Cell system (BioRad). After electrophoresis, gels were washed, and substrate buffer was added for 16 h at 37 °C. Subsequently, gels were washed and stained with Coomassie Brilliant Blue R‐250 (Sigma). The proteolytic activity of MMPs appeared as clear bands against a blue background of Coomassie Blue‐stained gelatin substrate. The gel was washed with distilled water and scanned using the GS‐800 Calibrated Densiometer (Bio‐Rad). MMP activity (MMP2 and MMP9) was quantified using densitometric analysis (Quantity One, Bio‐rad).

### 2.7 Immunofluorescence Analysis

Cell‐laden hydrogel discs were fixed with 4 wt.% paraformaldehyde (PFA, Sigma) in Hank's balanced salt solution (HBSS) (ThermoFisher) for 20 min, permeabilized for 5 min with 0.2% v/v Triton X‐100/HBSS and incubated for 1 h in 2 wt.% bovine serum albumin/HBSS to block unspecific binding. To analyze cell morphology (F‐actin cytoskeleton) samples were stained with flash phalloidin 647 (BioLengend, 1:100), and for phenotypic analysis samples were incubated with the epithelial‐marker anti‐rabbit E‐cadherin (Cell Signaling Technology 24E10, 1:200, ON at 4 °C) and the mesenchymal‐marker anti‐rabbit Fibronectin (Sigma F2006, 1:150, ON at 4 °C). To analyze the deposition of a laminin‐rich layer, samples were incubated with primary rabbit anti‐mouse laminin antibody (Sigma L9393, 1:50, ON at 4 °C). After incubation with primary antibodies, samples were incubated with goat anti‐rabbit secondary antibody Alexa Fluor 594 (Molecular Probes, Invitrogen, 1:1000, 1 h at RT) and nuclei were counterstained with DAPI (Sigma). Samples were mounted with VECTASHIELD Antifade mounting medium (Vector Laboratories) and analyzed by Confocal Laser Scanning Microscopy (CLSM, Leica SP5 or SP8).

### 2.8 Gene Expression Analysis by qRT‐PCR

MCF10A cells were retrieved from hydrogels upon dissolution with 0.05% w/v trypsin/50 mM EDTA solution, followed by centrifugation. RNA was extracted using the Quick‐RNA MiniPrep kit (Zymo Research), as recommended by the manufacturer. Subsequently, 500 ng of total RNA were reversed transcribed to single stranded cDNA using Takara cDNA synthesis kit. Quantitative Real‐Time PCR (qRT‐PCR) was carried out using source RNA for the target genes (Table S1, Supporting Information) (Applied Biosystems and Integrated DNA Technologies). Samples were run in triplicates in the ABI Prism 7000 Sequence Detection System under the following conditions: 95 °C for 20 sec, followed by 40 cycles at 95 °C for 3 sec and 60 °C for 30 sec. Data was analyzed by the comparative 2^(−ΔCT) method and normalized by their housekeeping gene. To display results in a heat map, no clustering was used and a Z‐score normalization was performed on the read counts across samples for each gene. To plot the heatmap, Z‐scores were computed on a gene‐by‐gene (row‐by‐row) basis by subtracting the mean and then dividing by the standard deviation.

### 2.9 Confocal Raman Microscopy

*Raman instrument and acquisition parameters*: Analyses were performed using a confocal Raman microscope (alpha300R+, WITec, Ulm, Germany). A 532 nm green solid–state laser light source (32 mW) was applied through a 63x/1.0 NA water immersion microscope objective lens (W Plan‐Apochromat, Zeiss, Oberkochen, Germany). Inelastically‐scattered light was collected through the objective and directed via a 10 μm diameter silica fiber, acting as a confocal pinhole, to a high‐throughput imaging spectrograph (UHTS 300, WITec, GmbH, Germany) using a 600 groove per mm grating equipped with a thermoelectrically cooled (−60 °C) back‐illuminated charge‐coupled device (CCD) camera. Raman spectra were acquired from 0 to 3600 wavenumbers (cm^−1^). In all cases, the excitation laser intensity was kept constant between sample scans.

*Raman chemical imaging of 3D cell cultures*: The 3D hydrogel cultures with cells in different EMT/MET states were PFA‐fixed and embedded in 1% w/v agarose (Sigma‐Aldrich, A0701‐25G) in a 35 mm ibidi dish with TBS/CaCl_2_ for immobilization in hydrated state during chemical imaging. Raman depth scans of 3D biospecimen datasets were performed by first locating the highest signal‐to‐noise ratio (SNR) laser focal plane for sample excitation at the sample surface, followed by continuous scan data acquisition through the depth of the sample. Using 63x water immersion objective, 50 µm deep Z‐stack scans were acquired with X‐Y step size of 1 µm, Z‐step size of 2 µm, and integration time of 0.15 to 0.3 s per voxel. Total image size was dependent on each acinus physical dimensions, ranging from 100–180 × 150‐250 µm, keeping X‐Y, and Z resolutions constant at 1 and 2 µm respectively.

*Spectral preprocessing and analysis of 3D Raman chemical image datasets*: All acquired Raman spectra were preprocessed in WITec ProjectFIVE 5.2 and Matlab following the same pipeline on a per pixel basis as established previously:^[^ ^23^ ^]^ cosmic ray removal (filter size: 4; dynamic factor: 4.1), setting minimum value in Rayleigh region (−150‐50 cm^−1^) to zero (detector dark current to zero), normalization setting the main water peak average value (3220–3420 cm^−1^) equal to one, matrix/medium background subtraction using a matrix alginate blank background spectrum and rolling circle baseline correction (shape size: 300) to remove any other non‐specific background signal artifacts. Additionally, organoid image stacks were subjected to SNR thresholding to remove all voxels where maxima in the high wavenumber region (2800–3100 cm^−1^) were less than 10‐fold the standard deviation observed across the baseline of Raman‐silent region (2200–2600 cm^−1^) to ensure only acceptable SNR spectral data (SNR >10) was included in the quantitative analysis. Finally, all spectra were cropped from 400–3100 cm^−1^. Volumetric analysis of the cell components was performed by integrating the peak area for different spectral features: 732–356 cm^−1^ (“Cytochrome C”), 771–797cm^−1^ (“DNA”), 994–1023 cm^−1^ (“Proteins), 1037–1052 cm^−1^ (‘Glycogen), 1121–1126 cm^−1^ (‘Glucose), 1580–1585 cm^−1^ (‘Phenylalanine”), 1622–1698 cm^−1^ (“Lipids), and 2820–3032 cm^−1^ (‘Total Biomolecule”).^[^ ^23^ ^]^ The collagen, laminin, and fibronectin images were generated by linear combination modelling using reference spectra from the pure components, as previously described in,^[^ ^23^ ^]^ using 1 mg ml^−1^ Albumin unit spectrum. Finally, the z‐stack of biomolecular distribution maps were loaded into ICY 2.0.3.0 for rendering of 3D quantitative chemical images. Multivariate statistical analysis (t‐SNE) was carried out with MATLAB R2022a (MathWorks, Inc., United States), ignoring the biological “silent region” (1800‐2700 cm^−1^) to focus analysis on biochemical spectral regions of interest.

### 2.10 Production of Decellularized ECM from Mammary Fibroblast Cultures

Human mammary fibroblasts (FIB) were used to produce cell‐derived ECM from control (ndECM) and TGF‐β1 activated cultures (adECM). For fibroblast activation (aFIB), cells were seeded at 2 × 10^4^ cells cm^−2^ in 6‐well plates and maintained in complete media for 24 h, followed by serum starvation (0.1% v/v FBS, with addition of 0.2 mM ascorbic acid) for 24 h. Cells were then maintained for 3 days in medium supplemented with 10 ng/mL TGFβ1. For the control condition, cells were seeded in complete media at a density of 3.5 × 10^4^ cells/cm^2^ for 24 h followed by cultured in starvation medium for 4 days. Monolayers were washed with ddH_2_O for 10 min and then decellularized using the following scheme: 1 min incubation in 0.5% sodium deoxycholate (Sigma), followed by 3 min incubation in 0.5% v/v of Triton X‐100 with 0.15% v/v of NH_4_OH (Sigma), and finally 10 min in 25 U mL^−1^ of DNASE I (PanReac AppliChem). The obtained dECMs were processed for imaging or proteomics or washed with ddH_2_O, scrapped, freeze‐dried, and maintained at −80 °C until used for subsequent studies.

To demonstrate successful activation and decellularization, FIB and aFIB cultures (before and after decellularization) were processed for immunostaining and imaged, as previously described, but using the following primary antibodies: anti‐mouse aSMA (SigmaA5228, 1:400, ON at 4 °C), anti‐rabbit fibronectin (Sigma F3648, 1:150, ON at 4 °C) and anti‐rabbit Collagen Type I (Rockland 009‐001‐103, 1:100, ON at 4 °C). Fibronectin production was estimated from CLSM images of ndECM and adECM based on pixel intensity using Fiji by mean gray value quantification in selected regions. Matrix thickness was determined from CLSM Z‐stack images. Gene expression of FIB and aFIB was analyzed by qRT‐PCR as previously desribed (Table S1, Supporting Information).

### 2.11 Proteomic Analysis of adECM and ndECM

For proteomics analysis, ndECM and adECM were solubilized in 100 mM Tris pH 8.5 with 1% sodium deoxycholate, 10 mM tris‐(2‐carboxyethyl)‐phosphine (TCEP) and 40 mM chloroacetamide for 10 min at 95 °C under agitation (Thermomixer‐Eppendorf, 1000 rpm). Each sample was processed following the solid‐phase‐enhanced sample‐preparation (SP3) protocol, as described in.^[^ ^24^ ^]^ Enzymatic digestion was performed with Trypsin/LysC (2 micrograms) ON at 37 °C at 1000 rpm. Protein identification and quantitation was performed by nanoLC‐MS/MS. This equipment was composed by an Ultimate 3000 liquid chromatography system coupled to a Q‐Exactive Hybrid Quadrupole‐Orbitrap mass spectrometer (Thermo Scientific, Bremen, Germany). Samples were loaded onto a trapping cartridge (Acclaim PepMap C18 100Å, 5 mm x 300 µm i.d., 160 454, Thermo Scientific) in a mobile phase of 2% ACN, 0.1% FA at 10 µL mi^−1^n. After 3 min loading, the trap column was switched in‐line to a 50 cm by 75 µm inner diameter EASY‐Spray column (ES803, PepMap RSLC, C18, 2 µm, Thermo Scientific, Bremen, Germany) at 250 nL mi^−1^n. Separation was generated by mixing A: 0.1% FA, and B: 80% ACN, with the following gradient: 5 min (2.5% B to 10% B), 120 min (10% B to 30% B), 20 min (30% B to 50% B), 5 min (50% B to 99% B) and 10 min (hold 99% B). Subsequently, the column was equilibrated with 2.5% B for 17 min. Data acquisition was controlled by Xcalibur 4.0 and Tune 2.11 software (Thermo Scientific, Bremen, Germany). The mass spectrometer was operated in data‐dependent (dd) positive acquisition mode alternating between a full scan (m/z 380–1580) and subsequent HCD MS/MS of the 10 most intense peaks from full scan (normalized collision energy of 27%). ESI spray voltage was 1.9 kV. Global settings: use lock masses best (m/z 445.12003), lock mass injection Full MS, chrom. peak width (FWHM) 15s. Full scan settings: 70k resolution (m/z 200), AGC target 3e6, maximum injection time 120 ms. dd settings: minimum AGC target 8e3, intensity threshold 7.3e4, charge exclusion: unassigned, 1, 8, >8, peptide match preferred, exclude isotopes on, dynamic exclusion 45s. MS2 settings: micro‐scans 1, resolution 35k (m/z 200), AGC target 2e5, maximum injection time 110 ms, isolation window 2.0 m/z, isolation offset 0.0 m/z, spectrum data type profile.

Raw data was processed using Proteome Discoverer 2.5.0.400 software (Thermo Scientific) and searched against the UniProt database for the *Bos taurus* and *Homo sapiens* proteomes and the NIST human spectral library. A common protein contaminant list was also considered. The MSPepSearch and Sequest HT search engines were used to identify tryptic peptides. The ion mass tolerance was 10 ppm for precursor ions (both software) and 20 ppm / 0.02 Da for fragment ions (MSPepSearch and Sequest HT, respectively). Maximum allowed missing cleavage sites was set to 2. Cysteine carbamidomethylation was defined as constant modification, while methionine oxidation, protein N‐terminus acetylation, Met‐loss and Met‐loss+acetyl were defined as variable modifications. Peptide confidence was set to high. The processing node Percolator was enabled with the following settings: maximum delta Cn 0.05; decoy database search target False Discovery Rate‐FDR 1%, validation based on q‐value. Protein‐label‐free quantitation was performed with the Minora feature detector node at the processing step. Precursor ion quantification was performed at the processing step with the following parameters: only the unique plus razor peptides were considered, precursor abundance was based on intensity, and normalization was based on the total peptide amount.

To determine differentially expressed proteins (adECM vs ndECM), at least two unique peptides had to be identified for a protein to be included in the analysis. For the selection of upregulated proteins, the adECM/ndECM ratio was set to FD ≥ 1.5; and for downregulated proteins it was set to FD ≤ 0.67, with an adjusted *p*‐value set to ≤ 0.05. Heatmap was performed on heatmapper.ca software. Volcano plot analysis was performed with the GraphPad Prism 8.0 software version 8.01.

*Bioinformatics analysis*: The results obtained were further treated according to the method described by *Kulej et al*.^[^ ^25^ ^]^ Proteins were compared to the matrisome project^[^ ^26^ ^]^ to identify ECM‐related proteins. Gene Ontology (GO) annotation, biological processes (BP), and KEGG analyses were performed on differentially expressed proteins on the Search Tool from EnrichR. Uregulated proteins were also analysed using MSigDB Hallmark 2020^[^ ^27^ ^]^ and DAVID Bioinformatics^[^ ^28^ ^]^ databases.

### 2.12 Preparation of Hybrid dECM‐ALG Hydrogels

The freeze‐dried dECMs were solubilized by enzymatic digestion with 2 mg mL^−1^ of pepsin (Sigma Aldrich, 1:10 ratio) in 0.02 M HCl for 24 h at 37 °C under agitation (Thermomixer‐Eppendorf, 400 rpm). Samples were then neutralized with 0.2 M NaOH. The dECM‐ALG hydrogels were obtained by mixing solubilized dECM with RGD‐ALG solution, at a final concentration of 1.6 wt.% ALG and 1.2 ± 0.3 mg mL^−1^ dECM. The mixture was maintaining under agitation ON at 4 °C to ensure homogenous distribution of both components. For crosslinking, the dECM‐alginate gel‐precursor solution was mixed with nanoCaCO_3_ (Fluka, molar CaCO_3_/COOH = 1) and GDL (molar CaCO_3_/GDL = 0.125) to trigger gelation. Hydrogel discs were casted between Teflon plates as previously described. To visualize both components in hybrid hydrogels, the dECM was labelled with Alexa Fluor 647 NHS Ester (Thermofisher, 1:100, ON at 4 °C and 400 rpm), and coumarin‐grafted blue‐fluorescent ALG (λex/em: 404/477 nm) previously synthesized by our group were used.^[^ ^29^ ^]^

### 2.13 3D Culture in Hybrid dECM‐Alginate Hydrogels

To establish 3D cultures in hybrid hydrogels, MCF10A cells were added to the gel precursor mixture before crosslinking and hydrogel discs were formed as described in section 2.12. To evaluate the effect of matrix composition on epithelial cell behavior, cells were cultured in soft (G´≈200 Pa) ALG (blank), ndECM‐ALG (to mimic the composition of normal breast tissue) or adECM‐ALG (to mimic the composition of breast tumors) hydrogels. To evaluate the effect of matrix stiffness, cells were cultured in soft adECM‐ALG hydrogels, which were dynamically stiffened after 1 day of culture, through secondary ionic crosslinking. For this, cell‐laden hydrogels were washed with HBSS and incubated in 50 mM of BaCl_2_ for 15 min. After washing, fresh media was added and renewed after 1 h. The viscoelastic properties of soft and stiffened hybrid hydrogels were analyzed by oscillation rheometry. Metabolic activity, gene expression, immunostaining analysis, and imaging were performed as described in previous sections.

### 2.14 Statistical Analyses

Statistical analyses were performed using GraphPad Prism Software (GraphPad Software Inc., version 8.01). Results comparing 2 groups were compared using t‐test or Mann‐Whitney in case of parametric or non‐parametric distributions, respectively. Results comparing more than 2 groups were compared using Kruskal‐Wallis test or Wilcoxon test in case of non‐parametric distributions paired or unpaired, respectively. All analysis were performed with a confidence level of 99% and statistical differences were represented by *(*p* < 0.05), **(*p* < 0.01), ***(*p* < 0.001) and ****(*p* < 0.0001).

## 3 Results

### 3.1 RGD‐ALG Hydrogel Facilitates Epithelial Morphogenesis and Enables EMT/MET Induction

We started by showing that normal mammary epithelial cells form acini in ALG hydrogels without undergoing spontaneous EMT but remain responsive to their microenvironment. For this, MCF10A cells were cultured in ALG hydrogels modified with cell‐adhesive RGD peptides with stiffness akin to the normal mammary gland (G’≈ 200 Pa),^[^ ^30^ ^]^ a formulation previously optimized by our group.^[^ ^10^, ^22^ ^]^ Different degrees of EMT and MET were induced by treating cells with TGF‐β1 (**Figure** **1**A). We established four distinct cell populations along the EMT/MET spectrum: an untreated epithelial‐like state (E), two TGFβ1‐induced E‐to‐M mesenchymal‐like states (EM1 and EM2: 3 and 14 days with TGFβ1, respectively), and one reversed M‐to‐E state (ME: 7 days with TGF‐β1 followed by 7 days without). In all conditions, hydrogel stiffness remained practically unchanged throughout culture (Figure 1B‐i) and cells remained metabolically active (Figure 1B‐ii). The EM1 and EM2 states presented slightly lower metabolic activity compared to control cells, which was expected as TGF‐β1 is a well‐known inhibitor of breast epithelial cells growth,^[^ ^31^ ^]^ but this effect was partially reversed in ME cells. As shown in Figure 1C, hydrogel‐embedded MCF10A cells were able to proliferate and form multicellular structures in all conditions. The non‐treated E cells organized into polarized acini with lumen and a basement membrane layer (Figure S1A, Supporting Information). These acinar structures exhibited high expression of E‐cadherin (E‐cad, epithelial marker) and no‐to‐low expression of fibronectin (FN, mesenchymal marker), showing that 3D culture in ALG hydrogels does not induce EMT. In contrast, TGFβ1‐treated cells (EM1 and EM2) formed more disorganized structures with heterogeneous sizes and invasive features, with decreased expression of E‐cad at the cell surface (see also Figure S1B, Supporting Information) and pericellular accumulation of FN within multicellular aggregates, suggesting the occurrence of EMT. Finally, TGFβ1‐treated cells that were further cultured without stimulation (ME) showed partial recovery of E‐cad expression (see also Figure S1B, Supporting Information), while retaining some FN expression, particularly within larger multicell aggregates, suggesting the occurrence of MET. The EM1 and EM2 states also showed higher expression of MMP2 and MMP9, as compared to E and ME, consistent with a migratory/invasive phenotype (Figure 1D). The morphological and molecular changes observed among the different states (E, EM1, EM2, ME) highlight the dynamic nature of malignant transitions and their impact on cell phenotype. Gene expression analysis (Figure 1E) further supported these differences, providing additional molecular evidence for the occurrence of EMT and MET (please refer to Figure S2 (Supporting Information) for the statistical differences). Compared to the E state, cells undergoing EMT (particularly EM2) exhibited decreased expression of epithelial markers, such as *OCLN* and *EPCAM* (p = 0.0195, and p = 0.003, respectively), increased expression of mesenchymal markers including *FN1*, *CDH2*, and *MMP2* (p = 0.0025, p = 0.0033, and p = 0.0166, respectively), as well as increased expression of EMT transcriptional factors, like *SNAI2* (p = 0.0234) and other EMT‐related genes, such as *ITGB4* (p = 0.0277). In the ME population, many of these alterations were partially reversed, with the overall expression profile showing a significant decrease of mesenchymal markers and a slight recovery of epithelial markers (please refer to Figure S2, Supporting Information for a more detailed overview). Principal component analysis (PCA) of the gene dataset of the different populations highlighted gene profile similarities between EM1 and EM2, as well as between E and ME (Figure 1F).

### 3.2 Cell Populations at Different EMT/MET States Present Distinct Biochemical Profiles

**Figure 2:**
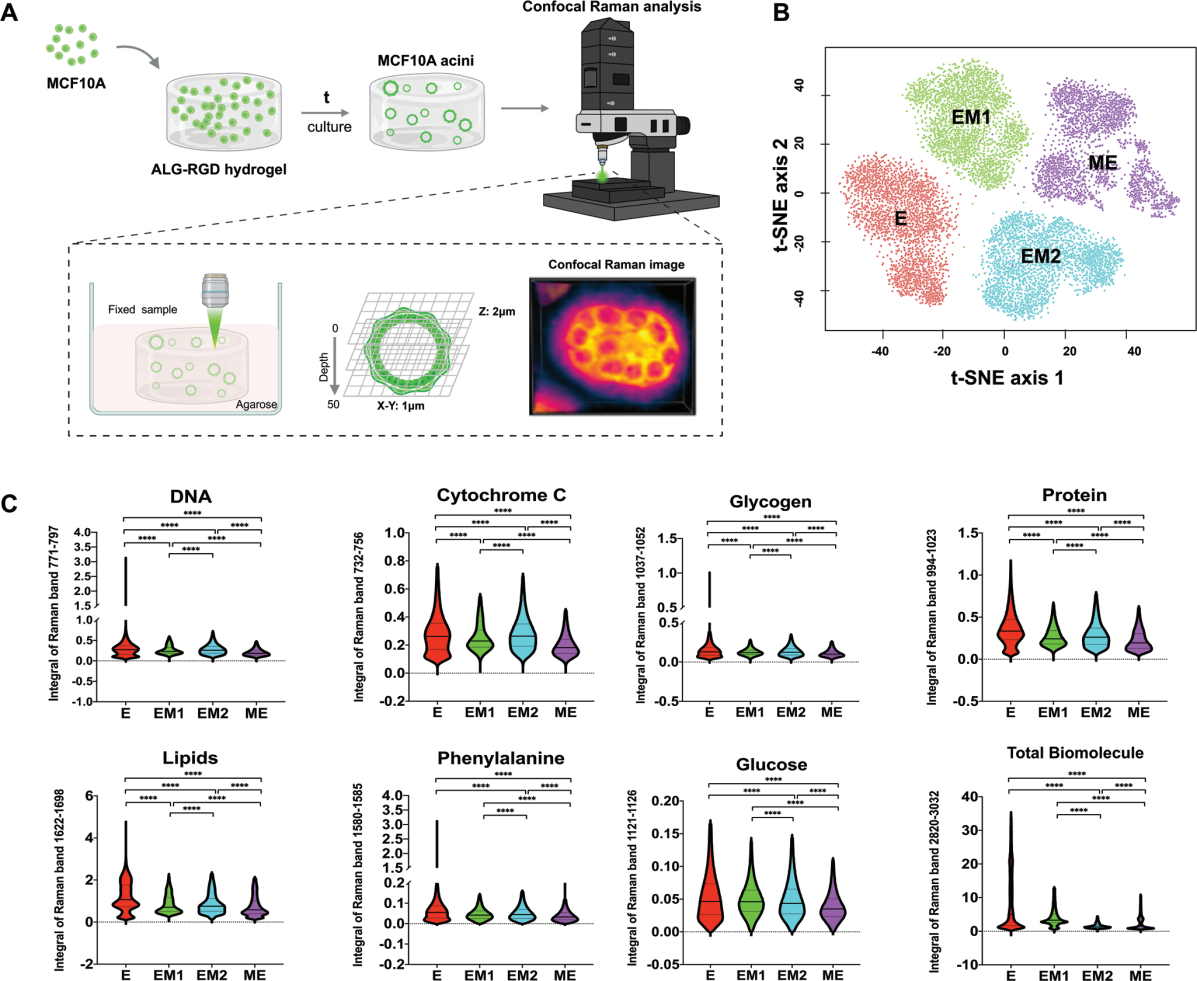
Cell populations at different EMT/MET states present distinct biochemical profiles. A) Schematic representation of the Confocal Raman analysis work‐flow: after culture, whole‐mounted samples of E, EM1, EM2 and ME states were fixed, embedded in 1% agarose and directly imaged for quantitative chemometric phenotyping. B) Multivariate separation of acini‐like structures organoids spectra via t‐SNE depicts the differences among the four different EMT/MET states (E in red, EM1 in green, EM2 in blue, ME in purple). Data represents 1 single experiment, additional data for 3 individual experiments is presented in Figure S2C (Supporting Information). n = 3 spheroids per group). C) Volumetric analysis of Raman specific band intensity for different cell populations: Nucleic acids (771–797 cm^−1^), Cytochrome C (732–756 cm^−1^), Glycogen (1037–1052 cm^−1^), Proteins (994–1023 cm^−1^), Total Lipids (1622–1698 cm^−1^), Phenylalanine (1580–1585 cm^−1^), Glucose (1121–1126 cm^−1^) and Total Biomolecule (2820–3032 cm^−1^). Data refers to 3 individual experiments with several hundred‐thousand Raman spectra in each group (E: n = 373 275, EM1: n = 111 623, EM2: n = 336 399, ME: n = 370 649). Statistical analysis was performed using non‐parametric and unpaired Kruskal‐Wallis test (****p < 0.0001).

The biochemical profiles of the different EMT/MET populations were characterized by Confocal Raman spectral imaging (RSI), which enabled high‐content label‐free analysis of a wide range of biomolecules in hydrogel‐embedded acini, with minimal sample preparation. The experimental workflow is depicted in **Figure** **2**A, and detailed information on data processing is provided in.^[^ ^23^ ^]^ The four populations – E (red), EM1 (green), EM2 (blue), and ME (purple), were clearly distinguishable based on the analysis of the biochemical spectral features by t‐SNE (Figure 2B). Furthermore, univariate identification of sub‐cellular components was achieved using spectral band assignments of DNA (nuclei), Cytochrome C, Glycogen, Protein, Lipids, Phenylalanine, Glucose and Total Biomolecule (Figure 2C; Figure S3A, Supporting Information). Due to the many chemical species in common across these biological components, their Raman spectra appear quite similar even if there were statistically significant differences between the biochemical profiles of the four populations (Figure 2C).

**Figure 3:**
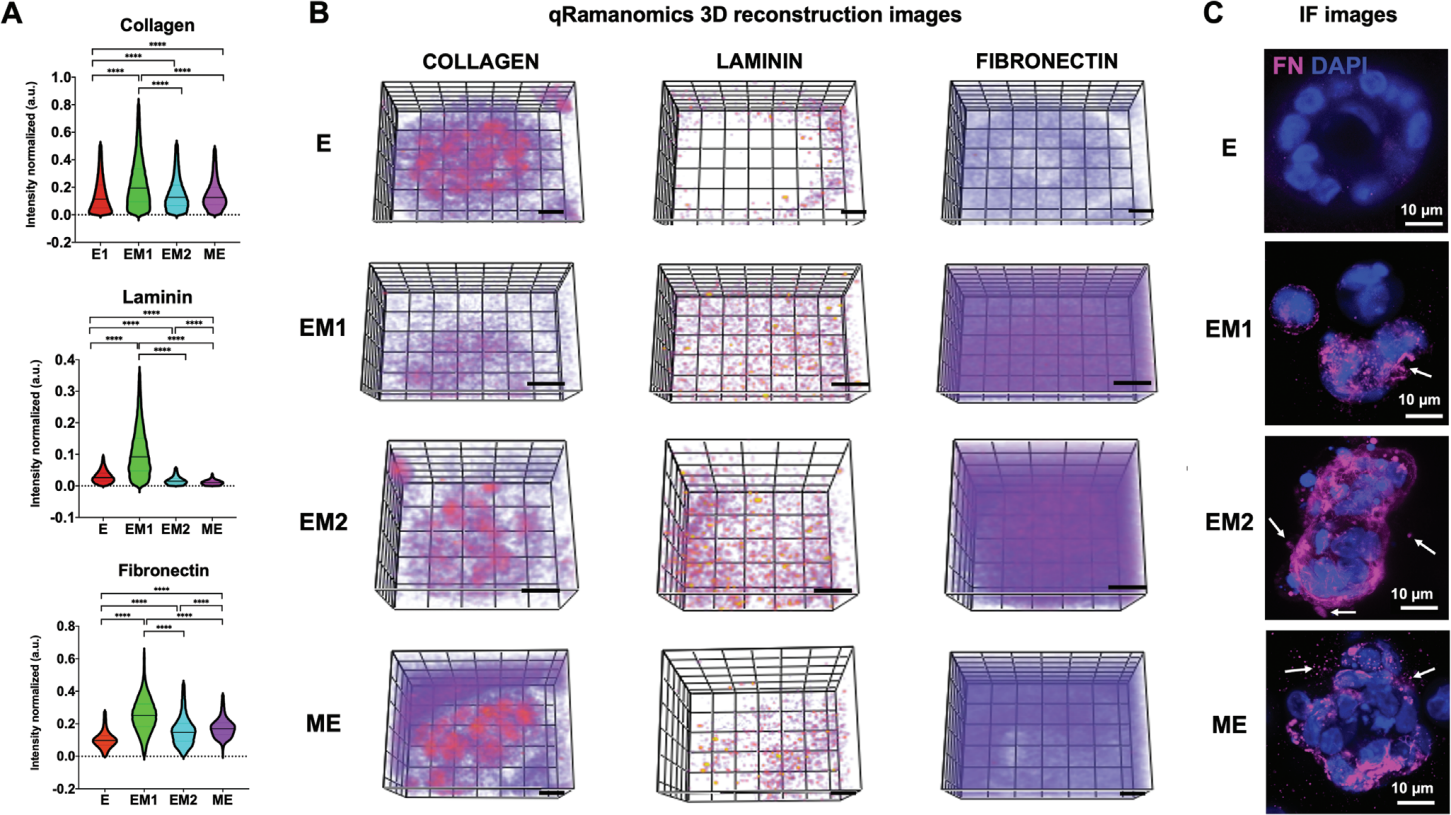
Quantitative qRamanomics reveals differences in the quantity and spatial distribution of ECM proteins across different EMT/MET states. A) Quantitative qRamanomics of Collagen, Laminin and Fibronectin (n  =  3 individual experiments). Statistical analysis was performed using non‐parametric and unpaired Kruskal‐Wallis test (****p < 0.0001). B) High‐content qRamanomics imaging of collagen, laminin and fibronectin. Scale bars, 10 µm. C) Representative immunofluorescence images of whole‐mounted samples of cells in E, EM1, EM2, and ME states stained for FN (magenta). Some FN deposition was also detected in the surrounding hydrogel matrix (white arrows). Please note that the two imaging techniques do not refer to the same sample.

qRamanomics was used for quantitative chemometric phenotyping as described in.^[^ ^23^ ^]^ This allowed direct structural and quantitative compositional characterization of specific biological components (collagen, laminin, and fibronectin) with subcellular spatial resolution. The quantitative qRamanomic (**Figure** **3**A) and the 3D reconstruction images (Figure 3B) revealed notable differences among the different EMT/MET states. Collagen and laminin delocalized from their initial acinar/peri‐acinar location and became randomly dispersed throughout the multicellular aggregate and the extracellular space, suggesting a disruption in the organization of the acini structures and their BM. The FN levels were significantly increased in the EM and ME states compared to the E state (Figure 3A and B) and the protein was not confined to cells but distributed within and around the acini. This expression pattern was corroborated by immunofluorescence (IF) analysis as previously shown in Figure 1 and shown at higher magnification in Figure 3C. The IF images further confirm that FN accumulates throughout the multicellular aggregate and can also be detected in the surrounding hydrogel matrix (white arrows).

### 3.3 Normal and Tumor‐Like dECM can be Generated in Vitro from the Same Fibroblast Source

**Figure 4:**
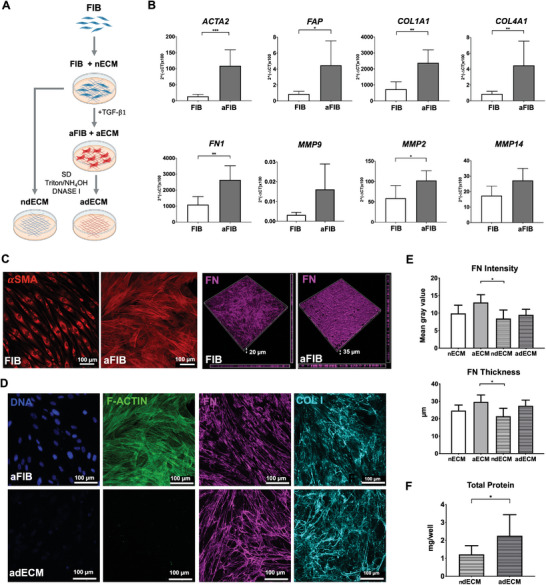
Cell‐derived dECM was obtained from TGFβ1‐activated and untreated fibroblasts. A) To produce adECM (tumoral) and ndECM (normal), monolayer cultures of mammary fibroblasts (FIB) were activated with TGFβ1 (aFIB) or left untreated (FIB), respectively, and then decellularized. Created with BioRender.com. B) The mRNA expression levels of activation markers (*ACTA2*, *FAP)*, ECM‐related genes (*FN1, COL1A1, COL4A1*), and remodeling‐related genes (*MMP2, MMP9, MMP14*) were all up‐regulated in aFIB compared to FIB (n = 5 individual experiments). C) CLSM images of control and activated monolayers, show higher expression of αSMA (red) and FN (magenta) in aFIB compared to FIB. D) CLSM images of native (aECM) and decellularized (adECM) monolayers (DNA in blue, f‐actin in green, FN in magenta, COL I in cyan), show that upon decellularization cellular and nuclear material were efficiently removed while ECM components were preserved. E) Thickness and pixel intensity of the FN layer in control versus activated cultures, before and after decellularization (n = 6 individual experiments). F) Total protein content in aFIB and FIB cultures. Data is presented as mean ± standard deviation. Statistical significance: *p < 0.05, **p < 0.01, ***p < 0.001.

Next, we produced cell‐derived dECM under controlled in vitro conditions, which was later combined with RGD‐ALG hydrogels to create hybrid dECM‐ALG hydrogels. As shown in **Figure** **4**A, normal mammary fibroblasts (FIB) were activated with TGF‐β1 to induce differentiation into CAFs.^[^ ^20^ ^]^ The resulting ECM‐rich monolayers were then decellularized to generate a surrogate of the tumoral ECM (adECM), and the dECM obtained from untreated fibroblasts was used as a control (ndECM). The TGFβ1‐treated fibroblasts (aFIB) showed significant up‐regulation of activation‐related genes, namely *ACTA2*, which encodes for smooth muscle alpha‐2 actin, and *FAP*, which encodes for fibroblast activation protein alpha, and genes related to ECM production (*FN1, COL1A1*, and *COL4A1*) and ECM remodeling (*MMP2, MMP9 and MMP14*) (Figure 4B). Higher production of αSMA in stress fibers and accumulation of secreted FN further supported successful fibroblast activation (Figure 4C). The decellularization of aFIB and FIB monolayers resulted in the efficient removal of cellular components (F‐actin) and nuclear material (DNA), while preserving key ECM proteins such as FN and COL I, which retained a well‐defined fibrillar structure (Figure 4D: adECM, Figure S4 (Supporting Information): ndECM). The pixel intensity and thickness of the FN layer were slightly higher in aFIB compared to non‐treated cultures, both before and after decellularization (Figure 4E). Correspondingly, a higher amount of total protein was retrieved from adECM compared to ndECM (Figure 4F), indicating increased matrix production by aFIB compared to FIB.

### 3.4 The dECM from TGF**𝛃**1‐Activated Fibroblasts Partially Mimics the Composition of Breast Tumor Matrices

**Figure 5:**
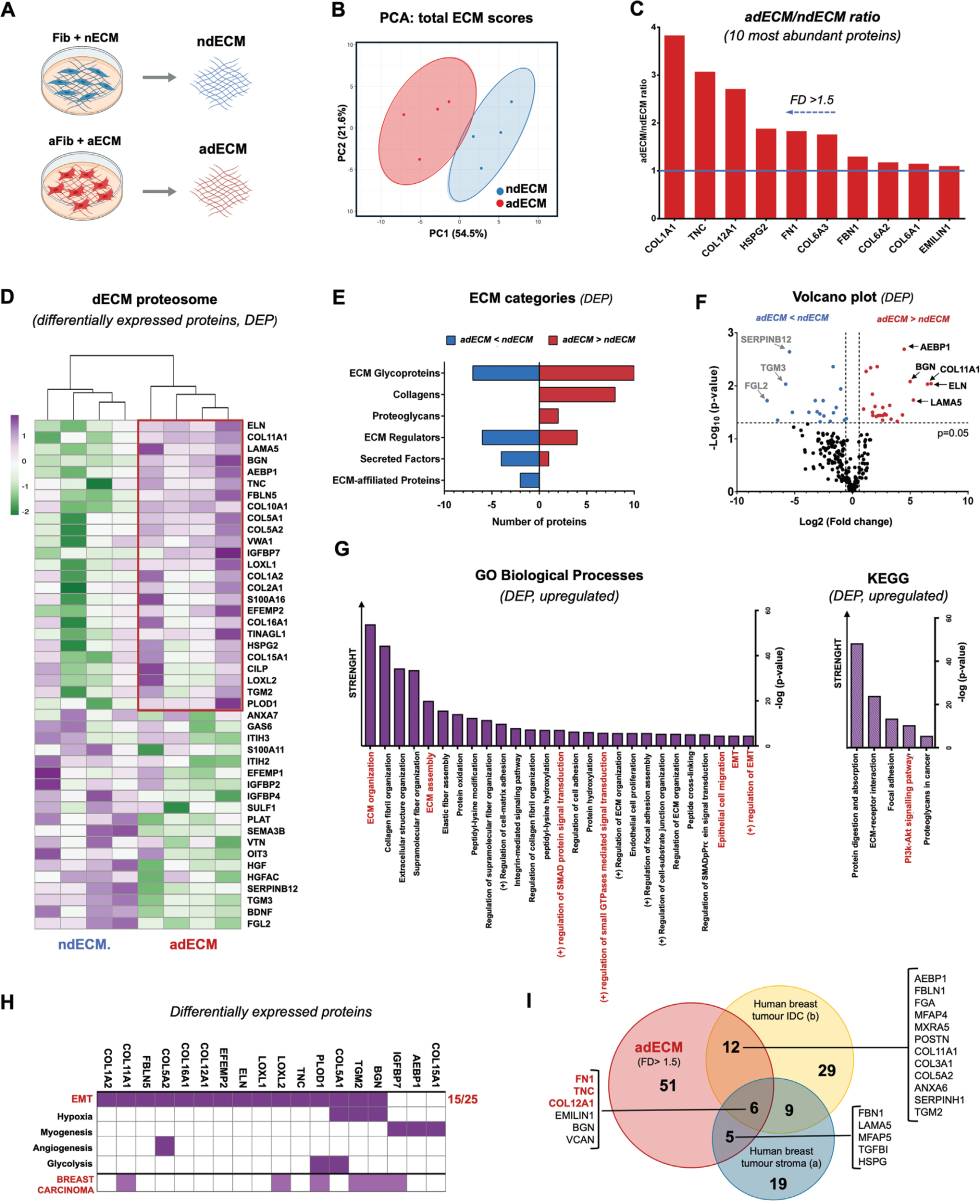
The dECM from TGFb1‐activated fibroblasts partially mimics the composition of breast tumor matrices. A) Decellularized ECM obtained from TGFβ1‐activated fibroblasts and non‐treated (control) fibroblasts was solubilized and analyzed by LC‐MS/MS proteomics (n = 4 biological replicates). Created with BioRender.com. B) Principal component analysis of differentially expressed proteins. C) 10 most abundant proteins in adCEM versus ndECM. D) Heatmap with unsupervised hierarchical clustering analysis showing 25 up‐regulated and 16 down‐regulated proteins in adECM versus ndECM (FD >1.5 and FD <0.67 p‐value < 0.05). E) ECM associated categories of up and downregulated proteins in adECM versus ndECM. F) Volcano plot displaying the distribution of all ECM associated proteins with relative protein abundance Log_2_(FD, adECM versus ndECM, x‐axis) plotted against the significance level (‐Log_10_(p‐value), y‐axis) and highlighting significantly (p‐value <0.05) up‐regulated (red) and downregulates (blue) proteins. G) GO enrichment analysis, showing related “Biological Processes” and “KEGG”, hierarchically organized by ‐log(p‐value) (*p*‐value < 0.05) from EnrichR. H) Upregulated proteins associated with cancer processes exported from MSigDB Hallmark 2020 database and mammary carcinoma from DAVID database. I) Venn diagram comparing up‐regulated proteins detected in adECM with matrix proteins from mammary carcinomas reported in 2 different studies: a)^[^ ^38^ ^]^ and b).^[^ ^32^ ^]^

The composition of the cell‐derived dECMs (**Figure** **5**A) was analyzed by quantitative matrisome‐targeted proteomics. The two‐dimensional scatter plot of the PCA of the protein dataset highlights the similarities among the 4 individual samples from the same type of matrix, and the differences between the two types of dECM (Figure 5B). The ten most abundant core‐matrisome proteins present in the adECM included fibrillar collagens (COL12A1 and COL6A2), tenascin, fibronectin, and perlecan (HSPG2), all of which have been previously reported to be overexpressed in breast tumors.^[^ ^32^, ^33^, ^34^ ^]^ A total of 44 differentially expressed proteins were identified in the adECM, where 25 were upregulated and 19 were downregulated compared to ndECM (Figure 5D). The upregulated proteins were mostly ECM glycoproteins, collagens and proteoglycans (Figure 5E). The Volcano plot (Figure 5F) shows the representation of the differentially expressed proteins in red (upregulated) and blue (downregulated) dots. The proteins displaying both large‐magnitude fold differences (FD >20, FD <0.03, x‐axis) and high statistical significance (p‐value < 0.05, y‐axis) are highlighted. The most upregulated proteins include AEBP1, BGN, COL11A1, ELN, and LAMA5, which have all been identified in breast tumors.^[^ ^32^, ^35^, ^36^, ^37^, ^38^ ^]^ The most downregulated proteins include SERPINB12, TGM3 and FGL2, which have been associated with tumor suppression.^[^ ^39^, ^40^, ^41^ ^]^

Figure 5G presents the GO terms for the most relevant biological processes and KEGG pathways associated with the upregulated proteins. The biological processes include ECM assembly, remodeling, and organization; collagen and elastic fibril organization; peptidyl‐lysine modifications; and cell‐matrix adhesion. These processes are integral to fibrotic tissue deposition, known as desmoplasia, which is a hallmark of breast tumors.^[^ ^3^, ^32^, ^38^, ^42^ ^]^ Other biological processes, such as positive regulation of epithelial cell migration and EMT, are associated with invasive potential and metastasis, and the regulation of SMAD proteins and small GTPases are intrinsically linked to the TGF‐β signaling pathway.^[^ ^43^ ^]^ The KEGG pathways include the activation of the PI3K‐Akt signaling pathway, recognized as a master regulator in cancer.^[^ ^43^ ^]^ The complete list of up/down‐regulated proteins is provided in the Supplementary Information (Figure S5A, Supporting Information).

The upregulated proteins in adECM were further analyzed using the MSigDB Hallmark 2020 database, revealing their association with several cancer‐related processes, including angiogenesis (1/25), glycolysis (2/25), myogenesis (3/25), hypoxia (3/25), apoptosis (3/25), and, most notably, EMT (15/25). The complete list is provided in the Supplementary Information (Figure S5B, Supporting Information). Additionally, analysis with the DAVID database indicated that 6 out of 25 of these proteins are associated with breast carcinoma (Figure 5H). We also compared the composition of the adECM with clinical samples from breast tumors. Specifically, we compared all the proteins present in the adECM with a FD >1.5 (relative to ndECM) against proteomics data of matrix‐associated proteins identified in human breast cancers. The Venn diagram (Figure 5I) shows that the adECM shares 27% of all ECM proteins upregulated in breast tumors stroma^[^ ^38^ ^]^ and 32% of those upregulated in invasive ductal carcinomas.^[^ ^32^ ^]^ Additionally, there was a global overlap of six proteins across all three datasets, including FN, TNS, and COL12A, which are among the most abundant proteins found in the adECM (Figure 5C). This highlights their widespread presence and importance in breast cancer. Notably, these proteins are part of a four‐protein signature that defines metastatic outcomes, as proposed in.^[^ ^32^ ^]^ The other protein in this signature, THBS‐2, was also detected in the adECM, though at a lower FD of 1.25. Collectively, our proteomics data strongly suggest that the adECM derived from CAF‐like TGF‐β1‐activated fibroblasts partially mimics key compositional features of the breast tumor matrix.

### 3.5 Hybrid dECM‐ALG Hydrogels Combine Tunable Composition and Stiffness

**Figure 6:**
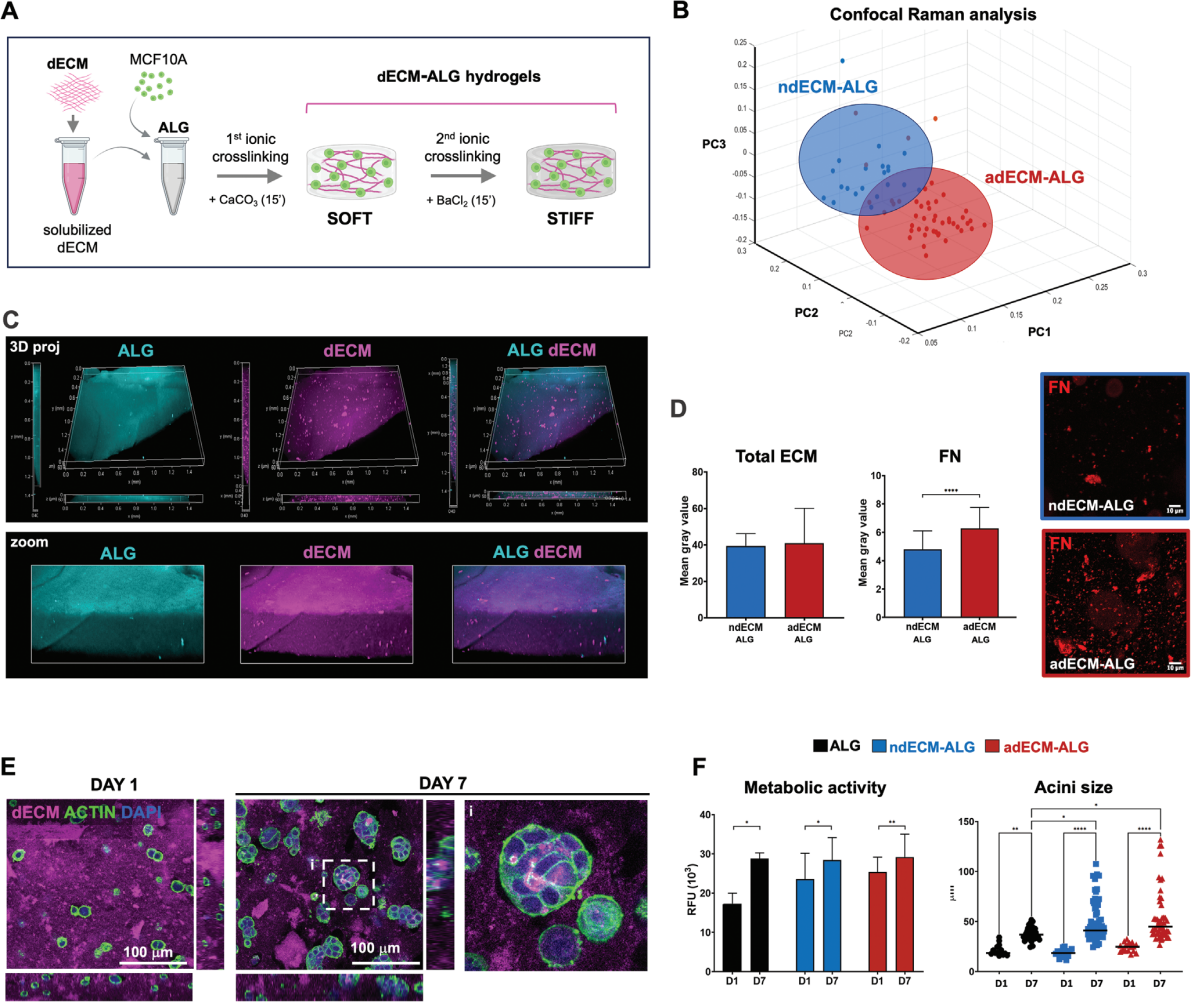
Hybrid ECM‐ALG hydrogels combine tunable composition and stiffness. A) Schematic representation of dECM solubilization and combination with ALG hydrogel to produce dECM‐ALG hybrid hydrogels via internal ionic crosslinking with Ca^2+^, followed by in situ stiffening via external ionic crosslinking with Ba^2+^. Created with BioRender.com. B) Multivariate separation of adECM‐ALG and ndECM‐ALG confocal Raman spectra data sets via PCA. (Data represents n > 20 spectra for each sample). C) CLSM images of fluorescently labelled dECM‐ALG hydrogels (dECM in magenta, ALG in cyan). D) Total ECM and FN content in ndECM‐ALG and adECM‐ALG hydrogels. E) CLSM images of MCF10A cells cultured in adECM‐ALG hydrogels after 1 and 7 days of culture (dECM in magenta, F‐actin in green, similar results were obtained in ndECM‐ALG hydrogels). F) Metabolic activity (resazurin assay) and acini size quantification for MCF10A cells cultured in dECM‐ALG hydrogels for 1 and 7 days, Data is presented as mean ± standard deviation. Statistical significance: *p < 0.05, **p < 0.01,***p < 0.001, ****p<0.0001.

To create the hybrid dECM‐ALG hydrogels, cell‐derived ECM (ndECM or adECM) was solubilized with pepsin^[^ ^17^ ^]^ and then combined with the RGD‐modified ALG gel‐precursor solution, followed by internal Ca^2+^‐mediated crosslinking with CaCO_3_/GDL (**Figure** **6**A). To increase the stiffness of cell‐laden dECM‐ALG hydrogels without altering their composition and structure, these were subjected to external ionic crosslinking in the presence of Ba^2+^. The 2D PCA plot of confocal Raman spectra data sets (Figure 6B) demonstrates a clear distinction in the biochemical composition between the adECM‐ALG and ndECM‐ALG hydrogels. Fluorescently labelled ALG (cyan) and dECM (magenta) were used to assess the spatial distribution of both components within dECM‐ALG hydrogels (Figure 6C). The confocal images reveal that dECM is distributed throughout the ALG hydrogel matrix, indicating the formation of an interpenetrating hybrid network.^[^ ^15^ ^]^ Additionally, some dECM‐rich foci were observed, suggesting microscale phase separation, likely due to the segregation and clustering of insoluble matrix components.^[^ ^44^ ^]^ The quantification of the total dECM present in the two types of hybrid hydrogels (Figure 6D) showed no significant differences, as antecipated. However, there was a significantly higher amount of FN in adECM‐ALG compared to ndECM‐ALG, which is in accordance with the proteomics results (Figure 5B). For 3D culture, MCF10A cells were incorporated into the dECM‐ALG gel‐precursor solution before crosslinking. In the hybrid hydrogels, cells maintained the ability to form acini (Figure 6E; Figure S6A, Supporting Information) and remained metabolically active throughout the culture period (Figure 6F), similar to what was observed in ALG hydrogels. The acini in the hybrid hydrogels exhibited a slightly larger average diameter and a less uniform size compared to those in ALG hydrogels. The variation is likely due to the structural differences and more heterogeneous network of the hybrid hydrogels, where larger acini often form around dECM‐rich foci (Figure 6E; Figure S6A, Supporting Information).

### 3.6 Matrix Composition and Stiffness Jointly Modulate EMT in dECM‐ALG Hydrogels

**Figure 7:**
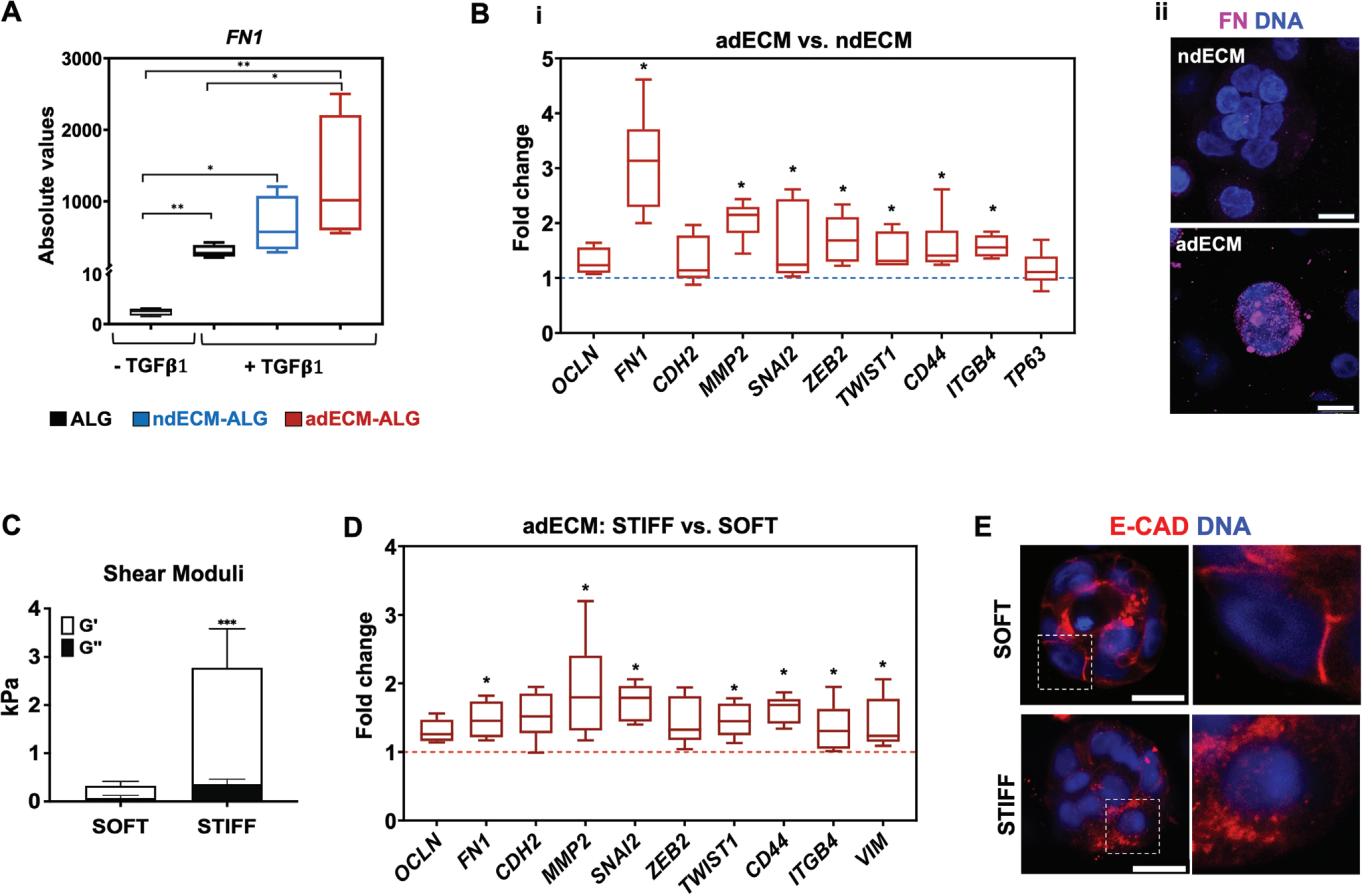
Matrix composition and stiffness jointly modulate EMT in dECM‐ALG hydrogels. A) Relative mRNA *FN1* expression by MCF10A cells cultured in ALG or dECM‐ALG hydrogels, with and without TGF‐β1 (data normalized to GAPDH, n = 4 individual experiments). B‐i) Relative mRNA expression of EMT‐related markers by MCF10A cells cultured in adECM‐ALG (red bars) compared to ndECM‐ALG hydrogel (blue line) (data normalized to GAPD, n =  6 individual experiments). B‐ii) Representative immunofluorescence images of dECM‐ALG hydrogels stained for FN (magenta, M‐marker). Scale bar: 10 µm. C) Viscoelastic properties of cell‐laden soft and stiff dECM‐ALG hydrogels after 1 day of culture (G’ and G”: elastic and viscous components of the shear moduli, respectively). Data are presented as mean ± standard deviation (n  =  3 individual experiments). D) Relative mRNA expression of EMT‐related markers of cells cultured in stiff (dark red bars) versus soft adECM‐ALG hydrogels (red line) (data normalized to GAPDH, n = 6 individual experiments). E) CSLM images of mammary acini stained for E‐cad (red) and DAPI (blue). Scale bar: 10 µm. Statistical significance: *p < 0.05, **p < 0.01, ***p < 0.001.

Using the dECM‐ALG hydrogels, we then evaluated the effect of matrix composition on the behavior of MCF10A cells when cultured in soft hydrogels (G' ≈200 Pa). The presence of ndECM did not significantly alter the expression levels of several EMT‐related genes (Figure S6B, Supporting Information) compared to ALG hydrogels, which served as a control. However, most of these genes were upregulated in the presence of adECM, confirming that incorporating dECM into ALG hydrogels induces a composition‐dependent effect on the embedded cells, as anticipated. Additionally, we compared the combined effects of matrix composition and TGF‐β1 signaling on *FN1* expression (**Figure** **7**A), the most significantly altered M‐marker in our experiments. While TGF‐β1 alone dramatically increased *FN1* expression in MCF10A cells cultured in ALG hydrogels (as also shown in Figure 1), the presence of adECM in hybrid hydrogels induced further upregulation. This suggests a synergistic contribution of altered matrix composition and TGF‐β1 signaling, both present in the breast TME, in inducing a malignant phenotype in mammary epithelial cells. Figure 7Bi shows the direct comparison between adECM‐ALG and ndECM‐ALG hydrogels, which only differ in composition being structurally similar. The presence of adECM in the hybrid hydrogel induced the upregulation of M‐markers (*FN1, MMP2)*, EMT transcription factors (*SNAI2, ZEB2, TWIST1*), and other EMT‐associated genes (*ITGB4, CD44*), suggesting the occurrence of a partial EMT. The higher expression of FN (Figure 7B‐ii) and SNAIL (Figure S6A, Supporting Information) have also been validated at protein level. These findings provide additional molecular evidence that adECM partially mimics the tumor matrix composition and that this altered composition contributes to EMT induction in mammary epithelial cells.

Next, we analyzed the behavior of MCF10A cells in stiffer adECM‐ALG hydrogels, as increased tissue rigidity is commonly associated with breast tumor development. The stiffness of the cell‐laden hybrid hydrogels was enhanced via external ionic crosslinking, resulting in an increase in G´ from approximately 200 Pa to 2500 Pa (Figure 7C), which falls within the range typically observed in breast tumors.^[^ ^30^ ^]^ This did not significantly affect cell metabolic activity (Figure S6D, Supporting Information). Cells in these stiffer adECM‐ALG hydrogels did not exhibit noticeable nuclear translocation of Yes‐associated protein 1 (YAP), a key mechanotransducer regulated by matrix rigidity. There were also no consistent alterations in the expression of YAP‐target genes *CYR61*, *CTGF*, and *ANKRD1* (Figure S6E), which had been previously observed by others.^[^ ^16^ ^]^ This suggests that alternative mechanotransduction mechanisms may be involved. Despite this, cells responded to the increased stiffness with significant upregulation of several M‐markers *(FN1, VIM, MMP2)*, EMT transcription factors *(TWIST1, SNAI2)*, and EMT‐associated genes *(CD44, ITGB4)* (Figure 7D), indicating a more pronounced mesenchymal shift compared to the effect of matrix composition alone. The delocalization of E‐cadherin from the cell membrane to the cytoplasm in stiffened hydrogels provides further evidence of EMT progression (Figure 7E) in these cells.

## 4 Discussion

The interplay between various components of the TME critically influences epithelial cells behavior and tumor progression. As the primary non‐cellular component of the TME, the ECM plays a pivotal role, with its altered biochemical and biophysical properties potentially driving EMT. Advanced 3D in vitro models that accurately recreate tumor cell‐matrix interactions are essential for uncovering related molecular mechanisms and screening new therapeutic strategies.^[^ ^45^ ^]^ This study introduces a novel hybrid 3D model that combines mechanically tunable alginate hydrogels with dECM derived from CAF‐like mammary fibroblasts, partially mimicking the composition of the breast tumor matrix, to investigate the effects of matrix properties on EMT.

We used a soft RGD‐modified ALG hydrogel as the bulk material, as it provides a neutral background for epithelial culture. Notably, our findings demonstrate that this simple, chemically defined hydrogel supports epithelial morphogenesis effectively, without the need for ECM proteins, which have been commonly incorporated in several studies.^[^ ^14^, ^15^, ^16^ ^]^ While mammalian cells cannot proteolytically degrade the dense, nano‐scaled mesh of alginate hydrogels, the soft RGD‐ALG 3D matrices exhibited sufficient plasticity to allow embedded cells to mechanically remodel their pericellular environment. This enabled the MC10FA cells to proliferate and develop into organotypic with apicobasal polarity, a hollow lumen, and a layer of endogenous basement membrane, resembling normal human mammary gland acini.^[^ ^10^ ^]^ Importantly, 3D culture in RGD‐ALG hydrogels does promote spontaneous EMT in normal mammary epithelial cells, as previously observed in other 3D models,^[^ ^10^ ^]^ but cells remain responsive to their microenvironment. By using TGF‐β1 to drive EMT and MET, we show that MCF10A cells can acquire intermediate and reversed phenotypes, exhibiting both epithelial and mesenchymal features, which illustrates that EMT is a continuum and not a binary process.^[^ ^1^ ^]^ The emergence of such hybrid states has been associated with metastatic processes, increased drug resistance, and poor patient outcomes.^[^ ^46^ ^]^ Although they represent a potential target for inhibiting metastasis, identifying these hybrid states in vivo remains challenging, as they are a snapshot of a cellular transition rather than a fixed phenotype. Therefore, our ability to generate intermediate EM and ME phenotypes in vitro, under controlled conditions, offers a valuable opportunity to better understand and characterize them. While we showed significant differences among the different EMT/MET states, the obtained cell populations displayed some heterogeneity, both at gene and protein levels, indicating that they were more likely to be a mixed culture of cells at different stages of EMT/MET. This is consistent with in vivo findings, as neoplastic cells from individual carcinomas often exhibit considerable plasticity and phenotypic heterogeneity, which may contribute to the appearance of more aggressive populations.^[^ ^47^ ^]^ To further characterize the different phenotypic states generated in ALG hydrogels, we used confocal Raman spectral imaging (RSI).^[^ ^48^ ^]^ The t‐SNE analysis, which evaluated the entire spectrum, clearly distinguished the four phenotypic states (E, EM1, EM2, and ME). This demonstrates the utility of the RSI technique in discriminating between cell populations at different EMT/MET states based on their unique biochemical signatures. Additionally, the use of qRamanomics for chemometric phenotyping^[^ ^23^ ^]^ enabled the direct quantitative characterization of the distribution of ECM proteins in epithelial acini, with subcellular spatial resolution. Interestingly, in EM and ME states, laminin and collagen were observed to delocalize from their initial position surrounding the acini. This suggests a breach of the basement membrane and disruption of acinar organization, a finding consistent with mesenchymal transitions in epithelial cells and a crucial step towards malignancy.^[^ ^49^ ^]^

While ALG hydrogels provide a well‐defined 3D environment for inducing EMT, they fall short in replicating the complex composition of the tumor ECM. To address this, we produced cell‐derived dECM under controlled in vitro conditions, which was then combined with RGD‐ALG to create hybrid hydrogels as more physiologically relevant 3D models. The dECM was obtained from mammary fibroblasts, one of the most abundant cell types in the breast TME, which were pre‐activated with TGF‐β1, a key mediator of the transition of normal resident fibroblasts to CAFs, as suggested by different studies.^[^ ^20^ ^]^ CAFs display characteristics of myofibroblasts, including increased expression of markers such as FAP, FSP1, and αSMA, as shown herein, and possess a unique ability to remodel their ECM. Through the process of desmoplasia, CAFs synthesize and secrete large amounts of new matrix components, while degrading neighboring ECM using proteases, such as MMPs.^[^ ^21^ ^]^ Our experimental model of FIB activation with exogenous TGF‐β1 effectively reproduced these features, indicating the successful induction of a CAF‐like phenotype. The adECM produced by TGFβ1‐activated fibroblasts showed significant compositional differences compared to the ndECM from untreated fibroblasts, as confirmed by proteomics. Our data revealed that many of the upregulated proteins in the adECM have been previously identified in breast tumors,^[^ ^32^, ^35^, ^36^, ^37^, ^38^ ^]^ and are involved in cancer‐related biological processes and signaling pathways.^[^ ^50^ ^]^ These processes include ECM organization and remodeling, which are characteristic of the tumor ECM's increased density, crosslinking, enzymatic alterations, and altered composition, that in turn influence tumor cell phenotype and invasiveness.^[^ ^3^ ^]^ Others are linked to abnormal epithelial behaviors such as increased migration and EMT. Notably, we observed approximately 30% overlap between the upregulated proteins in the adECM and matrix components identified by proteomics in clinical samples of different human breast tumors.^[^ ^32^, ^38^ ^]^ Considering the high variability among tumor tissues and the fact that our dECM is produced only by fibroblasts, while the tumor ECM involves contributions from various cell types, this overlap is significant. Overall, our proteomics data suggest that the adECM derived from CAF‐like TGF‐β1‐activated fibroblasts partially mimics key compositional features of the breast tumor matrix.

We then used dECM‐ALG hydrogels as a 3D model to evaluate the impact of matrix properties on EMT, where matrix composition and stiffness could be independently tuned. We first showed that while presenting a more heterogenous network that ALG hydrogels, as anticipated, the hybrid hydrogels also support MCF10A organization into acini. Then, we confirmed that the presence of a “normal” ndECM in soft hybrid hydrogels did not induce EMT, as observed in ALG hydrogels. Finally, we showed that the presence of adECM in hybrid hydrogels resulted in a significantly upregulation of mesenchymal genes and EMT‐related markers compared to ndECM‐ALG and ALG hydrogels. While TGF‐β1 showed to be a much potent EMT driver, inducing the acquisition of more marked mesenchymal phenotypes, our findings suggest that the composition of the tumor matrix has, per se, the potential to induce a partial EMT. Moreover, the presence of adECM in hybrid hydrogels also enhanced the effect of TGF‐β1 compared to ALG hydrogels. Interestingly, the ndECM also had a slight effect, likely due to the ability of certain matrix components to sequester soluble mediators, potentially enhancing the local activity of TGF‐β1 in the hydrogel‐embedded cells. However, this effect was more pronounced in the presence of adECM, confirming that the incorporation of dECM into ALG hydrogels has a composition‐dependent impact on epithelial cells, and that an altered “tumor‐like” adECM contributes more significantly to EMT progression.

Although there is limited literature available on the impact of breast tumor dECM on EMT, our findings are consistent with previous research. For example, decellularized human breast cancer biopsies have been shown to induce EMT and drug resistance in tumorigenic MCF‐7 cells.^[^ ^51^, ^52^ ^]^ Similar effects were observed when evaluating the response of these cells to dECM from triple‐negative breast cancer (highly aggressive) compared to luminal‐A breast cancer (less aggressive).^[^ ^53^ ^]^ Furthermore, cell‐derived dECM from tumor‐associated murine fibroblasts induced a more invasive and mesenchymal‐like phenotype in tumorigenic MDA‐MB‐231 cells compared to dECM from normal fibroblasts.^[^ ^54^ ^]^ In our model, the adECM contained different components typically found in of breast tumors, which have been linked with EMT and/or cancer progression, as previously described.

After examining the effect of matrix composition in soft dECM‐ALG hydrogels, we investigated the combined impact of matrix stiffness. To achieve this, we subjected cell‐laden adECM‐ALG hydrogels to a secondary ionic crosslinking step using Ba^2+^. Unlike previous studies that commonly used Ca^2+^, which plays a crucial role in various physiological processes, we chose Ba^2+^ to avoid potential interference. As demonstrated in other alginate‐based systems, this approach enables the dynamic stiffening of the hybrid hydrogels in the presence of cells without increasing the polymer concentration or altering the 3D network's architecture and mesh size.^[^ ^55^ ^]^ Cells in these stiffened adECM‐ALG hydrogels (G´≈2500 Pa) showed upregulation of several EMT‐related genes compared to soft adECM‐ALG hydrogels (G´≈200 Pa). This finding aligns with previous studies that have demonstrated a correlation between increased stiffness and EMT induction.^[^ ^56^, ^57^, ^58^ ^]^ Notably, the involvement of classical YAP‐mediated pathways in mechanotransduction was unclear in our system. There was no definitive evidence of YAP nuclear translocation at the protein level at any time point during culture, and the expression of YAP‐target genes in stiff versus soft hydrogels was inconsistent, showing upregulation after one week and downregulation after two weeks (Figure S5B, Supporting Information). While several studies showed these genes to be implicated in EMT, namely in breast epithelial cells and tumors, their regulation can be YAP‐independent. Also, recent studies have indicated that mechanotransduction can happen independently of YAP in MCF10A cells cultured in alginate‐based 3D models, as well as in human breast tumor samples, suggesting a context‐dependent role for YAP.^[^ ^16^ ^]^ Other studies showed that matrix stiffness can drive EMT in a YAP‐independent manner, namely via TWIST1‐regulated mechanotransduction pathways.^[^ ^58^ ^]^ Our observation of *TWIST1* upregulation in stiff versus soft hybrid hydrogels supports this possibility.

In summary, our findings indicate that the combination of aberrant matrix composition and increased stiffness, hallmarks of breast tumors, synergistically drives EMT in mammary epithelial cells. Further studies are necessary to elucidate the effects of specific matrix components and the mechanotransduction pathways involved. However, our results underscore the importance of considering both matrix composition and mechanical properties in EMT progression, highlighting the advantages of tunable dECM‐ALG hydrogels as a versatile 3D model for cancer research.

## 5 Conclusion

Hydrogels combining ALG and mammary fibroblast‐derived dECM offer a versatile in vitro platform for exploring the impact of matrix cues on EMT in the context of breast cancer. The bulk ALG hydrogels provide a neutral background for epithelial culture and allow for dynamic modulation of matrix stiffness, while the cell‐derived dECM partially captures the specific composition of both normal and tumor ECM. This hybrid 3D model enables matrix composition and stiffness to be independently tuned, effectively simulating the dynamic microenvironment of healthy and tumor breast tissue. The findings described in this study provide valuable insights into the role of an aberrant matrix in EMT induction in mammary epithelial cells, enhancing our understanding of these processes. The model's versatility extends to the use of dECM from other cell types, broadening its applicability. In cancer research, this platform may serve as a valuable tool for testing new ECM‐targeted therapies aimed at preventing or slowing EMT and cancer progression.

## Conflict of Interest

MMS invested in, consults and conducts sponsored research funded by companies related to the biomaterials field. The authors wish to acknowledge that Xiaoyu Zhao is affiliated with Supervision Medicine. The company has in no way been involved in the design, execution, or reporting of the research presented in this paper and has no ownership or rights to any data presented within this manuscript.

## Author Contributions

P.B.S. designed and carried out all the experiments, acquired and analyzed data, and wrote the manuscript. V.L. and P.B.S. developed and optimized confocal Raman microscopy analytical methods. X.Z. and V.L. acquired all Raman data, performed Raman data preprocessing and analysis. S.J.B. contributed to the acquisition and analysis of data concerning modulation of alginate stiffness. D.S.N. contributed to proteomics data organization and analysis. B.N.L. contributed to the design of the decellularization protocols. J.P. co‐supervised the work. M.M.S. supervised the Raman study and provided conceptual advice and financial support regarding the Raman experiments. C.C.B. designed and supervised the whole work, analyzed the data and wrote/revised the manuscript. The authors wish to acknowledge that Xiaoyu Zhao was affiliated with Supervision Medicine. The company has in no way been involved in the design, execution, or reporting of the research presented in this paper and has no ownership or rights to any data presented within this manuscript.

## Supporting information

Supporting Information

## Data Availability

The data that support the findings of this study are available from the corresponding author upon reasonable request.
