# Supplementary material for: Tunable Hybrid Hydrogels of Alginate and Cell‐Derived dECM to Study the Impact of Matrix Alterations on Epithelial‐to‐Mesenchymal Transition
Source: Adv Healthc Mater. 2024 Sep 9;13(29):2401032. doi: 10.1002/adhm.202401032 (PMC11582509; doi:10.1002/adhm.202401032)
Supplement: Supplementary file 1 — Supporting Information [file ADHM-13-0-s001.docx]

**SUPPORTING INFORMATION**

**Tunable hybrid hydrogels of alginate and cell-derived dECM to study the impact of matrix alterations on epithelial-to-mesenchymal transition**

P. Barros da Silva ^a,b,c^, Xiaoyu Zhao ^g,h^, Sílvia J. Bidarra ^a,b^, Diana S. Nascimento ^a,b,d^, Vernon LaLone ^f,g,h^, Bianca N. Lourenço ^a,b,c^, Joana Paredes ^a,e,i^, Molly M. Stevens ^f,g,h*^, C. C. Barrias ^a,b,d*^

^a^ i3S - Instituto de Investigação e Inovação em Saúde, Universidade do Porto, Rua Alfredo Allen 208, 4200-135, Porto, Portugal

^b^ INEB - Instituto de Engenharia Biomédica, Universidade do Porto, Porto, Portugal

^c^ FEUP - Faculdade de Engenharia da Universidade do Porto, Porto, Portugal

^d^ ICBAS - Instituto de Ciências Biomédicas Abel Salazar, Universidade do Porto, Porto, Portugal

^e^ IPATIMUP - Instituto de Patologia e Imunologia Molecular da Universidade do Porto, Portugal

^f^ Department of Materials, Imperial College London, Exhibition Rd, London, SW7 2AZ, UK

^g^ Department of Bioengineering, Imperial College London, Exhibition Rd, London, SW7 2AZ, UK

^h^ Institute of Biomedical Engineering, Imperial College London, Exhibition Rd, London, SW7 2AZ, UK

^i^ FMUP - Faculdade de Medicina da Universidade do Porto, Porto, Portugal

**Table S1 –** mRNA IDT probes.


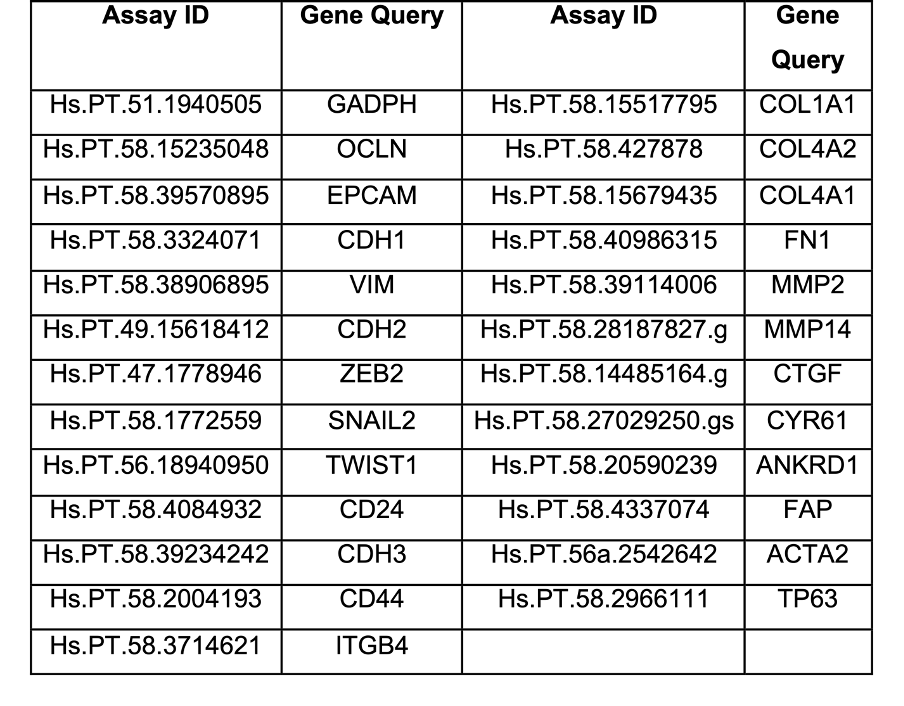


**
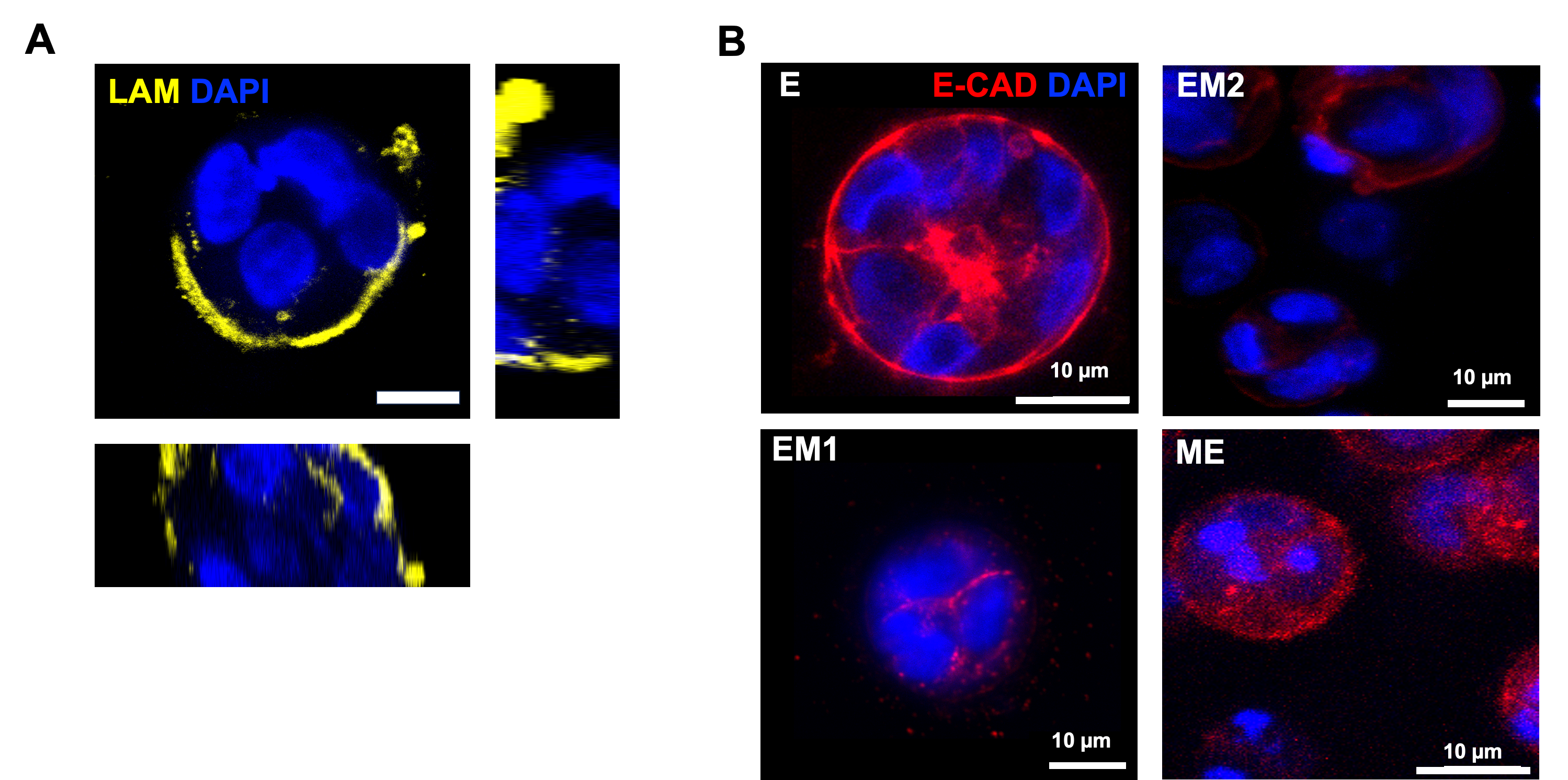
**

**Figure S1 –** **Fully defined RGD-alginate 3D matrices supported epithelial morphogenesis**. **A)** CSLM orthogonal projections in xy and yz of Laminin (yellow), showing the formation of a basement membrane-like layer around the acinar structures. Scale bar 10μm. **B)** Representative immunofluorescence images of whole-mounted 3D cultures at E, EM1, EM2 and MT states stained for E-cadherin (red, E-marker). Scale bar 10μm.


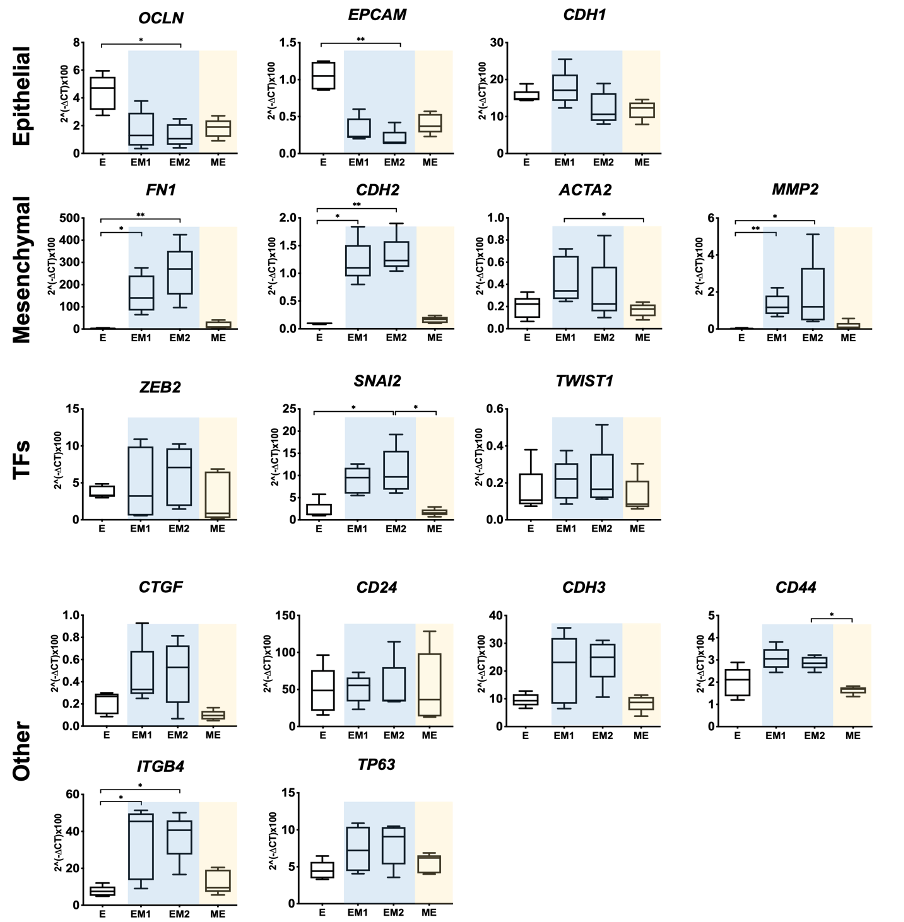


**Figure S2 –** **Fully defined RGD-alginate 3D matrices allow controlled EMT/MET induction**. **A)** mRNA expression profiles of the different EMT/MET states (Data normalized to GAPDH, n=5, Statistical significance: *p < 0.05, **p < 0.01).

**
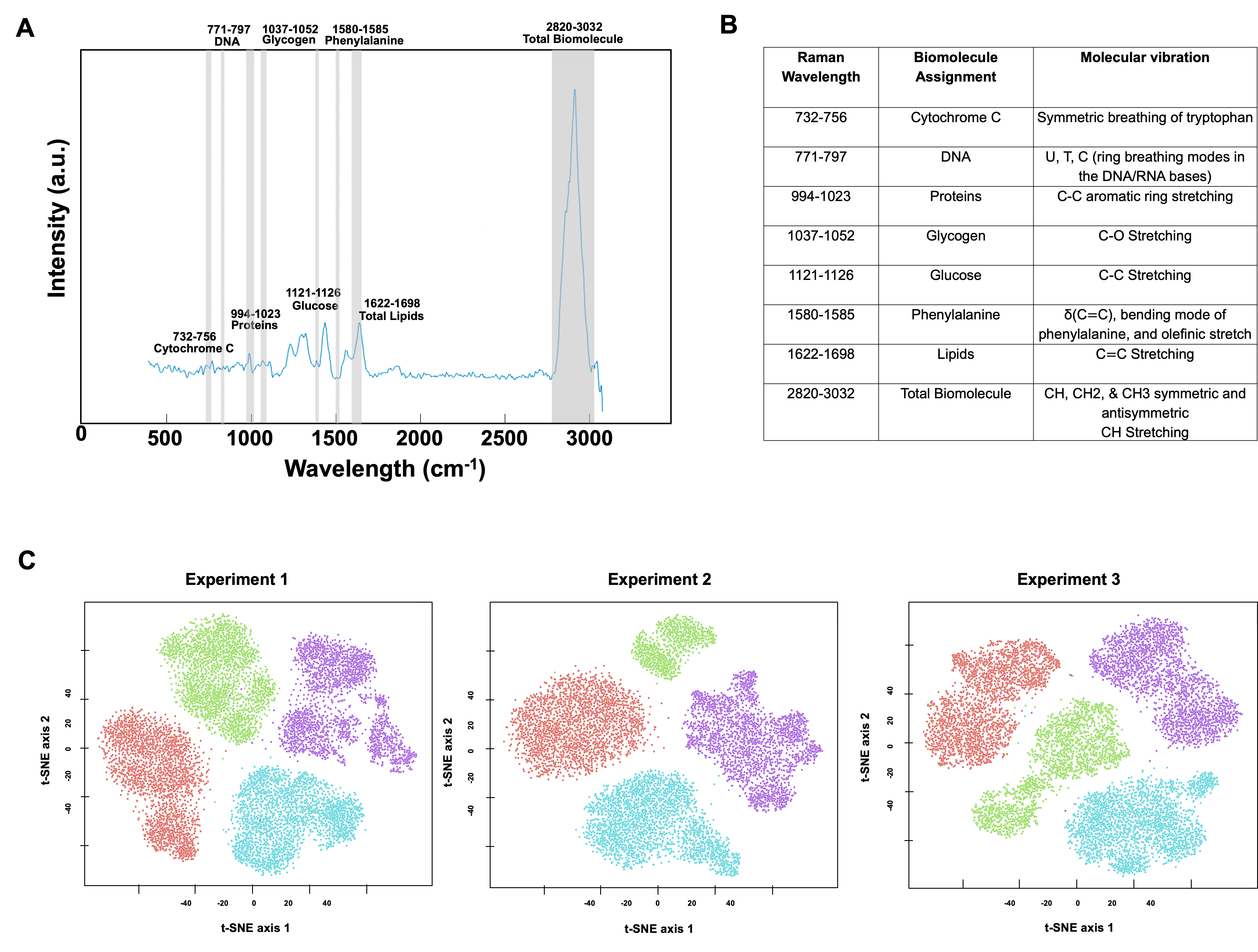
**

**Figure S3 –** **Cells in different EMT/MET stages present distinct biochemical signatures. A)** Example Raman spectra with highlighted Raman wavelength for the analysed cell components and **B)** their specific molecular vibration. **C)** Multivariate separation of acini-like structures organoids spectra via t-SNE depicts the differences among the four different EMT/MET states (E in red, EM1 in green, EM2 in blue, ME in purple) for three independent experiments.


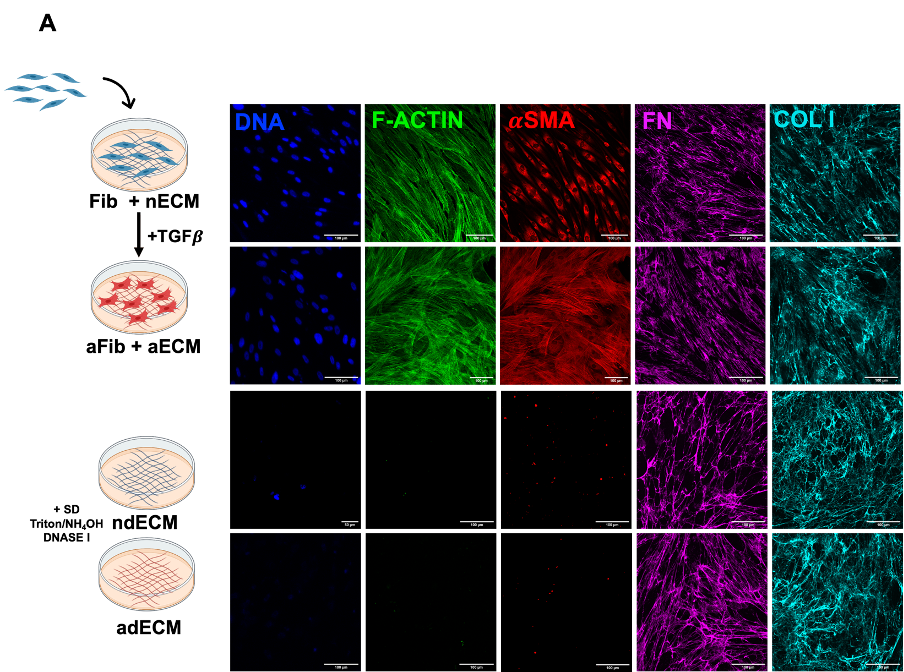


**Figure S4 –** **Generation of cell-derived dECM from normal and TGF-β1-activated human mammary fibroblasts. A)** CLSM images of control (FIB) and activation (aFIB) and decellularized ndECM and adECM monolayers, respectively (DNA in blue, F-actin in green, FN in magenta, COL I in cyan).


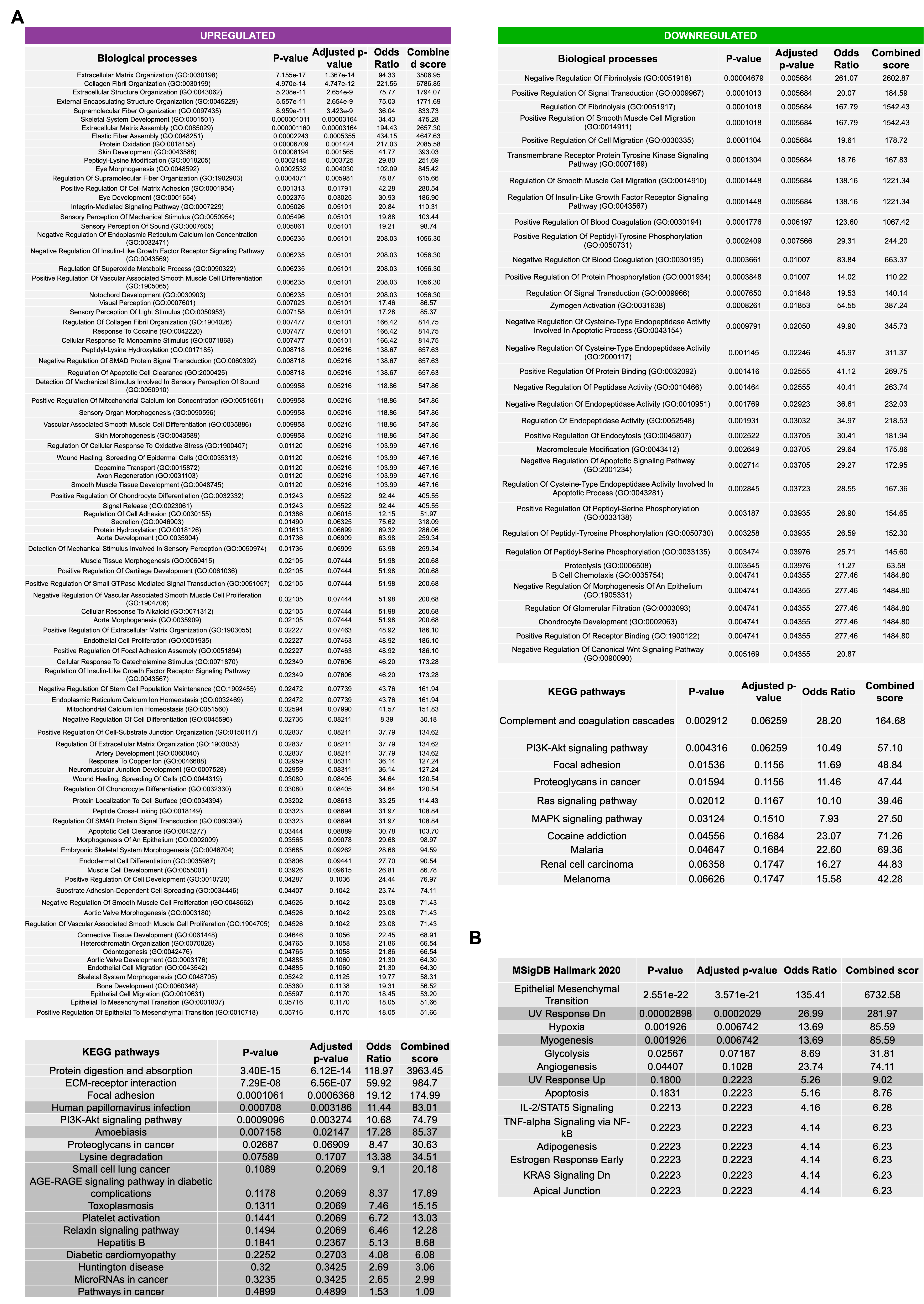


**Figure S5 – A)** Full list of GO enrichment analysis of “Biological Processes” and “KEGG” for proteins up-regulated and down regulated in adECM vs. ndECM, obtained from EnrichR. **B)** Full list of cancer processes associated with the upregulated proteins exported from MSigDB Hallmark 2020 database.


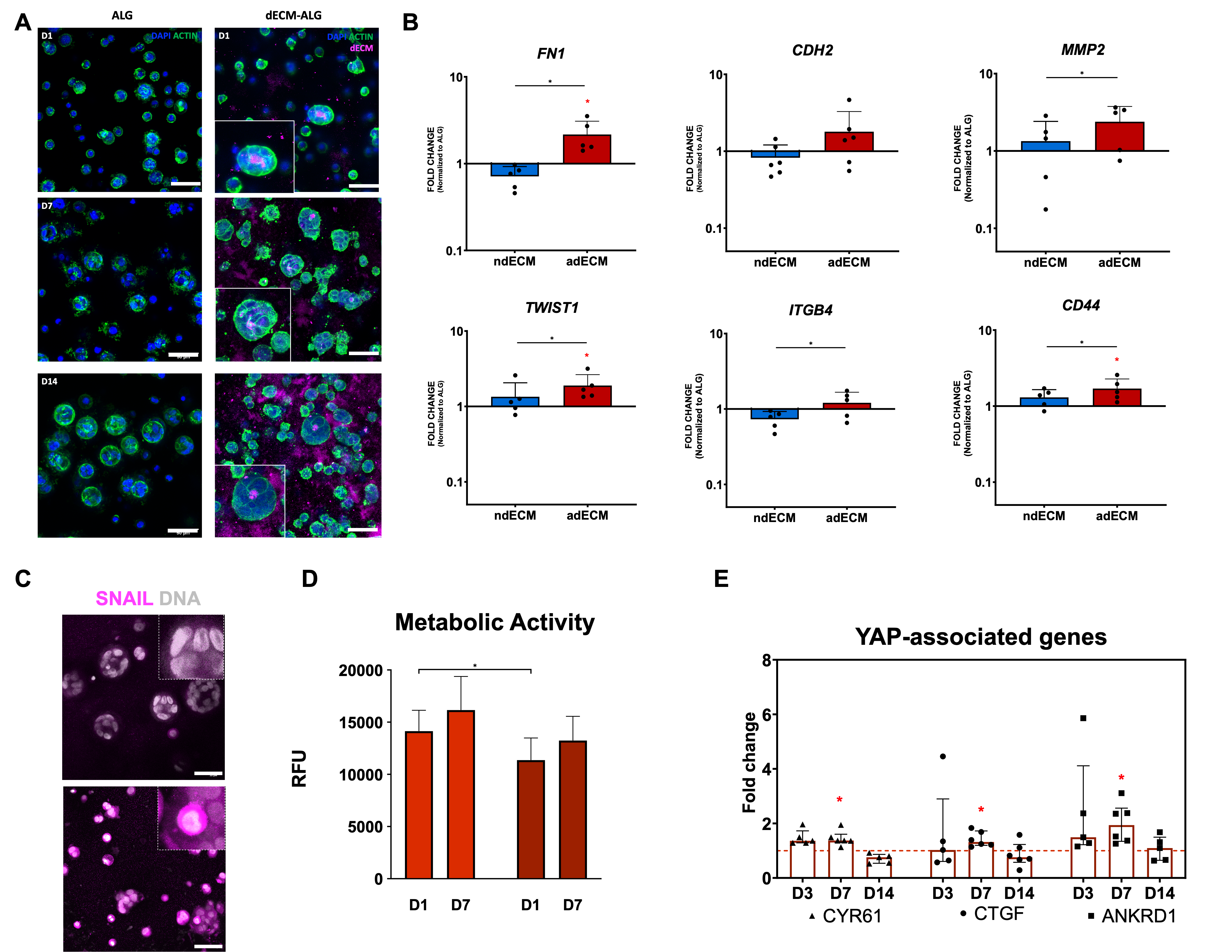


**Figure S6 –** **Hybrid ECM-ALG hydrogels combine tuneable biochemical and mechanical properties to modulate EMT in a TME-like microenvironment. A)** CLSM images of epithelial cells and organotypic structures after 1, 7 and 14 days of culture, in ALG and dECM-ALG hydrogels. **B)** mRNA expression of EMT-related markers of cells cultured in ndECM-ALG and adECM-ALG compared to ALG hydrogels (data normalized to GAPDH, n= 6. Statistical significance: (*) for ndECM vs adECM, (*) for ndECM or adECM vs ALG, p < 0.05) **C)** CLSM images of epithelial cells after 7 days of culture in dECM-ALG hydrogels stained for Snail (magenta). Scale bar 20μm. **D)** Metabolic activity of soft (red bars) and stiff (dark red bars) adECM-ALG hydrogels. **E)** Relative mRNA expression of YAP-target genes: *CYR61*, *CTGF*, and *ANKRD1* of cells cultured in stiff vs. soft adECM-ALG hydrogels. (*p < 0.05)
